# Boundary line models for soil nutrient concentrations and wheat yield in national‐scale datasets

**DOI:** 10.1111/ejss.12891

**Published:** 2019-11-15

**Authors:** Richard M. Lark, Vincent Gillingham, David Langton, Ben P. Marchant

**Affiliations:** ^1^ School of Biosciences University of Nottingham Nottingham UK; ^2^ AgSpace Agriculture Ltd. Dorcan Business Village Swindon UK; ^3^ Origin Enterprises Dublin Ireland; ^4^ British Geological Survey Nottingham UK

**Keywords:** agricultural management, fertilization, modelling

## Abstract

In boundary line analysis a biological response (e.g., crop yield) is assumed to be a function of a variable (e.g., soil nutrient concentration), which limits the response in only some subset of observations because other limiting factors also apply. The response function is therefore expressed by an upper boundary of the plot of the response against the variable. This model has been used in various branches of soil science. In this paper we apply it to the analysis of some large datasets, originating from commercial farms in England and Wales, on the recorded yield of wheat and measured concentrations of soil nutrients in within‐field soil management zones. We considered boundary line models for the effects of potassium (K), phosphorus (P) and magnesium (Mg) on yield, comparing the model with a simple bivariate normal distribution or a bivariate normal censored at a constant maximum yield. We were able to show, using likelihood‐based methods, that the boundary line model was preferable in most cases. The boundary line model suggested that the standard RB209 soil nutrient index values (Agriculture and Horticulture Development Board, nutrient management guide (RB209), 2017) are robust and apply at the within‐field scale. However, there was evidence that wheat yield could respond to additional Mg at concentrations above index 0, contrary to RB209 guidelines. Furthermore, there was evidence that the boundary line model for yield and P differs between soils at different pH and depth intervals, suggesting that shallow soils with larger pH require a larger target P index than others.

**Highlights:**

- Boundary line analysis is one way to examine how soil variables influence crop yield in large datasets.
- We showed that boundary line models could be applied to large datasets on soil nutrients and crop yield.
- The resulting models are consistent with current practice for P and K, but not for Mg.
- Models suggest that more refined recommendations for P requirement could be based on soil pH and depth.

## 1 INTRODUCTION

Farmers commonly add the essential plant nutrients, potassium (K), phosphorus (P) and magnesium (Mg), to the soil in fertilizers and manures. These additions contribute to the stocks of nutrients retained by the soil, from which plants obtain most of their requirements, rather than directly from the added fertilizer (Cooke, 1982; Stockdale, Goulding, George, & Murphy, 2013). If farmers do not maintain adequate stocks of available nutrients in the soil then the yield of the crop may be limited and other nutrients, notably nitrogen, may be used less efficiently, increasing harmful losses to the environment (e.g., Duan, Shi, Li, Sun, & He, 2014). In the short term, P, K and Mg remain in plant‐available forms in soil, so the farmer can estimate fertilizer requirements to maintain, build or deplete stocks of available nutrients on the basis of calculated offtake in the crop. With regular soil sampling to monitor the situation, the farmer can therefore avoid nutrient deficiency, while avoiding the economic and environmental costs of over‐fertilization (Goulding, Jarvis, & Whitmore, 2008).

This approach to nutrient management has long been the basis for the “RB209” system for advice to farmers in the United Kingdom (AHDB, 2017; Defra, 2010). A critical part of RB209, and other advisory systems, is the definition of target concentrations of available nutrients in the soil that should be maintained in order to avoid nutrient limitations on crop yield. In the UK system nutrient index ranges are defined with respect to nutrient concentrations in standard extractions. These index values are based on experimental evidence from past field trials. In the current system they do not reflect known soil factors that might induce variations in the crop requirement at within‐ or between‐field scales. It is therefore to be expected that the general target index for a particular nutrient might be excessive in some circumstances and inadequate in others.

Large amounts of data on soil (from sampling and analysis) and on crop yield (from monitors on combine harvesters) are acquired by farm businesses to improve crop and soil management. There is growing interest in the potential of such “big data” sources to improve the management and efficiency of farm enterprises (e.g., Wolfert, Ge, Verdouw, & Bogaardt, 2017). These large datasets could be used for an empirical reappraisal of current target index values. However, whereas a field fertilizer trial is carefully designed to reduce the yield variation induced by limiting factors other than the controlled treatment, at least within each block, and to ensure that this variation does not bias estimates of the fertilizer effect, this is not the case in an observational study of data from working farms. It is therefore necessary to find methods to characterize the dependence of crop yield on the concentrations in soil of nutrients of interest in datasets where other factors such as soil pH, non‐target nutrients in soil, soil depth, available water, weed competition and disease pressure, etc., vary in an uncontrolled way.

One quantitative model that has been used for this particular problem is boundary line analysis (BLA), first enunciated by Webb (1972), who proposed that the effect of some factor on the response of a biological system may be expressed by the upper boundary of the scatter plot of the response (variable *y* on the ordinate) against the factor (variable *x*_*i*_ on the abscissa). The boundary line is a function Λ(·), such that, for some value of *x*_*i*_ the largest value of the biological response is Λ(*x*_*i*_). The boundary line is interpreted as the response to variable *x*_*i*_ that is possible when other factors are not limiting.

Let us assume that there are *j* variables that are potentially limiting on *y*. We denote the *n*^th^ observation of the response by *y*_*n*_ and the corresponding set of potentially limiting variables take values *x*_*n*,*i*_,*i* = 1,…,*j*. The boundary function, Λ_*i*_, might be a limiting response to variable *x*_*i*_ in one of two senses. First, it might represent the response of variable *y* to *x*_*i*_ in a model that expresses von Liebig's (1863) “law of the minimum”:

(1) $$y_{n}\;=\;\min_{i\in \left\{1,\ldots ,j\right\}}\left[\Lambda_{i}\left(x_{n,i}\right)\right].$$

In words, the *n*^th^ instance of the response *y* is determined by the most limiting of the corresponding environmental variables, the variable for which the limiting response is smallest.

An alternative interpretation of the boundary response, as made by Elliot and de Jong (1993), is that it is a rate‐limiting function such that:

(2) $$y_{n}\;=\;y_{\max}\Pi_{i=1,\;\ldots \;,j}\left\{\Lambda_{i}\left\{x_{i}\right\}\right\},$$

where

(3) $$0\leq \Lambda_{i}\left(x_{i}\right)\;\leq 1\;\forall \;i,\;x_{i}\in \;\mathbb{R},$$

and where *y*_max_ is the largest possible response of the system, achieved only when

$$\Lambda_{i}\left(x_{i}\right)\;=\;1\;\forall \;i.$$

Note that the boundary function Λ_*i*_(·) will be represented by an upper limit on a scatter plot of *y* against *x*_*i*_ for some datasets only if the set contains a number of instances in which the *i*^th^ variable is limiting. The response of a crop to a nutrient, for example, will not correspond to an upper boundary on the scatter‐plot of yield against nutrient concentration if crop yield at all sites from which the data are obtained is limited by either available water or poor establishment. The dataset must therefore be large enough and cover sites with sufficient variation that there exists some non‐empty subset, ℬ_*i*_, of observation indices, such that:

(4) $$n\in {ℬ}_{i}\;\Rightarrow \;\Lambda_{i}\left(x_{n,i}\right)\;\leq \;\Lambda_{k}\left(x_{n,k}\right)\;\forall \;k\in \left\{1,\ldots ,j\right\}.$$

We say that a dataset in which some such subset exists *expresses* the boundary response.

The boundary line concept has been applied to quantify potentially limiting concentrations of crop nutrients in the soil on crop yield (e.g., Evanylo & Sumner, 1987; Kihara et al., 2016; Kihara & Njoroge, 2013; Wang et al., 2015: Tittonell & Giller, 2013). Shatar and McBratney (2004) used BLA to examine limiting effects of soil organic carbon, pH, K and Fe on the yield of sorghum based on a large dataset obtained with a yield monitor.

The outputs of a BLA can be used to define potentially limiting values of a variable as a guide to practice. For example, if growers ensure that concentrations of a soil nutrient are maintained within a range of values where the boundary line model is at maximum yield, then they reduce the risk that the nutrient will limit crop production (although other factors might). This was the approach taken by Walworth, Letzsch, and Sumner (1986) to define diagnostic norms for nutrient concentrations in plant tissue, and by Evanylo and Sumner (1987) and Evanylo (1990) to define target values for soil nutrients for soya bean and cucumber crops. This suggests that BLA could be a powerful way to exploit the information contained in large agronomic datasets for the refinement of target values for soil nutrient concentrations.

Milne, Ferguson, and Lark (2006) proposed a statistical model for data with a boundary line, in which the variate {*y*, *x*_*i*_} is modelled as a realization of a bivariate Gaussian random variate {*Y*, *X*_*i*_}, which is censored by the function Λ_*i*_(·), such that the underlying random variate is {min[*Y*, Λ_*i*_(*X*_*i*_)], *X*_*i*_} and the data are a realization of this random variate with the observed yield, including a Gaussian measurement error of mean zero and variance $\sigma_{e}^{2}$. The parameters of this model may be estimated by maximum likelihood, and this provides a basis for comparison with alternative models (with no censoring process) by means of likelihood‐based statistics such as Akaike's information criterion (AIC) (Akaike, 1973). It also allows the uncertainties in the estimated parameters of the boundary line to be quantified.

The approach of Milne et al. (2006) has been used in subsequent studies. Cossani and Sadras (2018) used it to examine the joint effects of nitrogen and water limitation on grain yields at a global scale, and Kindred, Milne, Webster, Marchant, and Sylvester‐Bradley (2015) used it to analyse results of experiments on the response of crops to applied nitrogen at the within‐field scale. Lark and Milne (2016) proposed a reparameterization of the model and used it to assess how the water‐filled pore space fraction of soils affects the potential rate of emission of nitrous oxide.

In this paper we use the method of Milne et al. (2006) and Lark and Milne (2016) to examine boundary line models for datasets on the yield of wheat and on concentrations of extractable P, K and Mg in the soil. The data were collected from subregions within arable fields in the UK for harvests in 2015, 2016 and 2017 and were originally collected as part of a commercial service to growers. We examine evidence for a boundary line model in each case and compare the results with recommendations to growers based on soil analyses under the RB209 system (AHDB, 2017; Defra, 2010). We look at how greater granularity in recommendations might be obtained by examining the boundary model for yield and soil P in soil subsets defined by pH intervals and soil depth.

## 2 MATERIALS AND METHODS

### 2.1 Soil and yield data

AgSpace Agriculture Ltd. conducts soil sampling for its customers on the basis of pre‐identified management zones within each field. These zones were the basic units for which soil and crop yield data were obtained in this study. The zones are map units, delineated in the field by experienced soil scientists, who made hand‐auger observations of the soil, paying particular attention to topsoil colour, topsoil and subsoil texture, stone content, carbonate content and soil depth. All these properties were assessed in the field following conventional soil survey practice; for example, inferring carbonate content from a “fizz” test with 10% hydrochloric acid (Hodgson, 1976). Note that the purpose of these observations was to allow the surveyor to assess the variability of the soil within the field and to identify distinct soil units to map. Any quantitative information on soil properties was obtained by subsequent sampling after the delineation of zones. Augering was not done on a fixed grid, but according to the surveyor's judgement, to allow the delineation of boundaries between contrasting soil map units by the interpretation of the auger observations in combination with observed slope, known geological boundaries, air photography and yield maps. This procedure corresponds to “free” soil survey (Dent & Young, 1981; White, 2006). The resulting soil map units are treated as management zones, to be managed on the basis of soil sampling undertaken within the units after they are delineated. It has been shown that soil map units, delineated by free soil survey, can account for the variation of soil properties and crop yield at the within‐field scale in the sense that the variation of these properties within the units is less than that within the field as a whole (King et al., 2005; Lark, Catt, & Stafford, 1998).

AgSpace Agriculture Ltd. then undertook soil sampling, structured by the zones. For the soil analyses reported here a total of 24 cores were collected per zone to a depth of 15 cm. The cores were collected in a “W” pattern across a zone and aggregated in the field to form one bulk sample for that zone. A subsample was then taken for laboratory analyses. The resulting data can be treated as estimates of the zone mean for each soil property (Webster & Burgess, 1984). In the work reported in this paper we consider available P, K and Mg following extractions, as required for comparison with RB209 Indices (MAFF, 1986). Details are given in the reference, but in summary the P measurement was Olsen P. Phosphorus was extracted from a 5‐ml sample of air‐dried soil in 100 ml of sodium bicarbonate buffered at pH 8.5 at a temperature of 20 ± 1^°^C. The concentrations of K and Mg measurements were from an extraction in M ammonium nitrate. A 10‐ml sample of air‐dried soil was extracted in 50 ml of the extractant. Soil pH was measured in a 1:2.5 soil:water suspension with a combination electrode and pH meter.

In each season the mean yield of combinable crops was extracted for each zone. The raw yield monitor data were subjected to preliminary editing to remove values that appeared to be influenced by movement of the combine (e.g., turning on headlands) or by partial fill of the cutter bar. The point yield values were then aggregated in each zone to give a zone mean yield.

The data used for this study were on yields of winter wheat and soil nutrient data aggregated to zone scale. All the available yield and soil data for this crop were extracted for analysis from the AgSpace database and used in the analyses described below. For harvests in 2015, 2016 and 2017 there were, respectively, 6,609, 5,954 and 4,541 zones in total from 240, 202 and 166 farms, respectively; an average of 27.5, 29.5 and 27.4 zones per farm in the respective seasons.

Summary statistics for zone yields and analytical results are presented in Appendix S1 (Tables S1 and S2), dividing the data into feed and milling wheat crops. Note that, in all cases, the nutrient concentrations showed a marked positive skewness, which was reduced to below 1.0 by transformation to natural logarithms. The data on yield did not require transformation.

### 2.2 The boundary line model

Details of the boundary line model based on a censored bivariate Gaussian random variable are given by Lark and Milne (2016). In summary, the model is a bivariate distribution of the observed response variable, *y*, and an independent covariate, *x*_*i*_. It is assumed that this distribution depends on a latent bivariate‐normal random variate **Z** = {*Y*, *X*_*i*_}^T^ with joint density function:

(5) $$f\left(y,x_{i}\right)\;=\;\phi_{2}\left(z|\mu ,C\right),$$

where *ϕ*_2_(⋅| **μ**, **C**) is the bivariate normal density function for a random variate with mean vector **μ** and covariance matrix **C**. The variate, **Z**, is censored by a boundary function Λ_*i*_(*X*_*i*_| **β**) with parameters in **β**, to give a censored variate $\bar{Z}={\left\{\bar{Y},X_{i}\right\}}^{T}$. In this paper we consider upper boundaries (a maximum yield is determined by the value of *x*), so:

(6) $$\bar{Z}=\;{\left[\min\left\{Y,\Lambda_{i}\left(X_{i}|\beta \right)\right\},X_{i}\right]}^{T}.$$

We assume, as in the general linear model, that the independent variable is known without error and that the observed response variable, *y*, is obtained by the observation of $\bar{y}$ with an observation error, which is normally distributed with mean zero and standard deviation *σ*_e_.

Lark and Milne (2016) derived the likelihood function for *y* conditional on *x*_*i*_ and some proposed set of parameters **β**, **μ**, **C** and *σ*_e_. If each of a set of *n* observations, $\left\{{\overset{˘}{y}}_{k},x_{i,k}\right\},\;k=1,\ldots ,n$,is treated as independent, one may then compute the negative log‐likelihood for a set of parameter values, given the observations, as:

(7) $$\ell \;=\;-\sum_{k=1}^{n}\log f_{b}\left({\overset{˘}{y}}_{k}|x_{i,k};\beta ,\mu ,C,\sigma_{e}\right).$$

The maximum likelihood estimate of model parameters was obtained by finding a set of values that minimize *ℓ* over the dataset.

### 2.3 Model fitting and assessment

#### 2.3.1 Fitting a boundary line model by maximum likelihood

We followed Milne et al. (2006) by using an approximation to the measurement error of the yield data, *σ*_e_, so as to reduce the computational demands of finding the ML estimates. This approximate value was set at the nugget variance for the variogram of yield data in each set. As the empirical variogram could be estimated for lag distances down to short, within‐field scales, and the support of the yield data is the whole zone over which the yield monitor data are aggregated, we expect the contribution of any sources of variation other than measurement error to this nugget to be small. Empirical variograms were estimated with the **variogram** function of the **gstat** package for the **R** platform (R Core Team, 2014) and variogram models were fitted by weighted least squares using the **fit.variogram** function in the same package.

Because the boundary line model of Lark and Milne (2016) is based on an underlying bivariate normal distribution for the yield and nutrient concentrations, it is necessary to decide whether this assumption is plausible. To do this we examined histograms of the data and summary statistics, evaluating these in the light of the possibility that the observed yield is subject to some right (upper) censoring. On this basis we chose to transform nutrient concentrations to natural logarithms for all data used in this study, but kept yield in tonnes per hectare.

Boundary line models, describing the limits of a dataset, are susceptible to the effects of outliers and all methods use some criterion to identify and remove them first. In this study we used the bagplot proposed by Rousseeuw, Ruts, and Tukey (1999) as a bivariate generalization of Tukey's (1977) boxplot. The univariate boxplot defines an interval (box) which includes half the observations, and then uses the spread of the box to define a wider set of fences outside of which data are regarded as outliers. The bagplot similarly defines a “bag” which contains the central half of the data and a fence around the bag obtained by expanding it by a factor. Values outwith the fence are regarded as outliers. In this study we followed this procedure using the **bagplot** procedure in the **aplpack** library for the **R** platform (R Core Team, 2014).

We used a simple boundary line model with a linear response to a variable *x*_*i*_ (nutrient concentration) bounded by a maximum yield:

(8) $$\Lambda \left(x_{i}\right)\;=\;\min\left[\beta_{0}+\beta_{1}x_{i},\beta_{2}\right],$$

where *β*_2_ is the maximum yield. The parameters of the boundary line model were therefore:

$$\beta =\left\{\beta_{0},\beta_{1},\beta_{2}\right\},$$

from Equation (8), **μ**, a vector including the mean values of the latent variables *y* and *x*_*i*_ and parameters of the covariance matrix, **C**, of the latent variables (their variances and correlation). For any specified values of these parameters, and with *σ*_e_ fixed at the nugget variance of the yield data, the negative log‐likelihood could be computed from the data with Equation (7). The **optim** procedure in **R** was used to find the values of the parameters that minimized the negative log‐likelihood. This procedure requires initial guesses of the parameters. After a first solution was obtained, we repeated the optimization, starting from this first solution and setting values for an optional scaling argument of the procedure using numerical partial derivatives of the likelihood at the first solution, such that a unit change in each model parameter produced a similar change in the likelihood. It is known that all numerical optimizations are prone to find solutions that are only locally optimal, so this procedure was repeated several times from different initial guesses, and the solution with the smallest negative log‐likelihood was selected.

#### 2.3.2 Comparing the boundary line model with alternatives

Two alternative models to the boundary line were also considered. The first is a simple bivariate normal distribution for which the parameters are just the mean values of yield and the variable of interest, their variances and correlation. This model was fitted by minimizing the negative log‐likelihood in the same way as for the boundary line model. This model would be appropriate if yield and the variable of interest, *x*_*i*_, were quite independent of each other (which would include the case where variable *x*_*i*_ is not limiting on yield for any of the observations) or if yield could be regarded as a linear function of *x*_*i*_ additive with effects of other independent variables. The second alternative was a bivariate normal distribution with a constant censor on yield. This model could be appropriate if some factor other than *x*_*i*_, and independent of it, limits yield, including general biophysical factors such as seasonal climate or disease pressure.

Evidence for the validity of the boundary line model can therefore be assessed by comparing its fit with that of the alternative models. The models may be compared with respect to their likelihood, but it is necessary to account for the number of model parameters. The bivariate normal model, for example, can be considered a special case of the boundary line model in which the parameters in **β** are such that no observations are limited by the boundary. For this reason, the negative log likelihood for the boundary line model can never be larger than that of the bivariate normal model. One way to compare models with respect to their likelihood while allowing for differences in their complexity is to compute Akaike's information criterion A (Akaike, 1973). If a model with *P* parameters is fitted and the minimized negative log‐likelihood is *ℓ* then:

(9) A=2ℓ+2P.

Over some set of alternative models we can minimize the expected Kullback–Leibler divergence between the estimated model and the process that generates the data by selecting the model for which A is smallest (Buckland, Burnham, & Augustin, 1997). Note that, in effect, the term 2*P* in Equation (9) is a penalty for model complexity. If the value of A for the *i*^th^ model in a set of *m* exceeds the minimum value of A over the set by Δ_*i*_ then one may compute the Akaike weight for that model as:

(10) $$w_{i}\;=\;\frac{\exp\left\{-\Delta_{i}/2\right\}}{\sum_{j=1}^{m}\exp\left\{-\Delta_{j}/2\right\}}.$$

The weight for the *i*^th^ model can be interpreted as the probability that the model is the best one for the variable, in the sense of having minimum Kullback–Leibler divergence, over the set of models considered (Buckland et al., 1997; Burnham & Anderson, 2004). In this study we used the Akaike weights for this purpose. We computed the weight for each of the three models considered for each dataset and examined the boundary line model if and only if its value of A was smallest in the set and its Akaike weight equalled or exceeded 0.5.

#### 2.3.3 Uncertainty in model parameters

The **optim** procedure can compute a Hessian matrix, **H**, for the model parameters. The element in the Hessian matrix for the *i*^th^ and *j*^th^ parameters, *β*_*i*_ and *β*_*j*_, is:

(11) $$H\left[i,j\right]\;=\;\frac{\partial }{\partial \beta_{i}\partial \beta_{j}}\ell^{2},$$

where *ℓ* is the negative log‐likelihood. In this case the covariance matrix of the model parameters, **∑**, can be estimated by the inverse of the Hessian matrix (Dobson & Barnett, 2018). The standard errors for each estimated parameter can be computed as the square root of the corresponding term on the main diagonal of **∑**.

In the case of the boundary line model used in this study, Equation (8), the independent variable *x*_*i*_ is potentially limiting on crop yield if *x*_*i*_ is less than a critical value, $x_{i}^{\text{crit}}$, beyond which the boundary line is constant. This critical value is given by:

(12) $$x_{i}^{\text{crit}}\;=\;\frac{\beta_{2}-\beta_{0}}{\beta_{1}},$$

where the terms are as defined for Equation (8). In order to assess the uncertainty in the value of $x_{i}^{\text{crit}}$, we assumed normal errors in the estimates of the boundary model parameters $\hat{\beta_{0}},\;\hat{\beta_{1}}$ and $\hat{\beta_{2}}$, and drew sample sets of these parameters from the multivariate normal random variable with distribution:

(13) $$N\left({\left\{{\hat{\beta }}_{0},{\hat{\beta }}_{1},{\hat{\beta }}_{2}\right\}}^{T},\sum \right).$$

This was carried out with the **mvrnorm** function from the **MASS** package for **R** (Venables & Ripley, 2002). A total of 500,000 subsets were drawn and $x_{i}^{\text{crit}}$ was calculated for each with Equation (12). The distribution of $x_{i}^{\text{crit}}$ was approximated by the empirical distribution from these samples. In particular, the 95% confidence interval was obtained. Because the distribution of $x_{i}^{\text{crit}}$ was not symmetrical, the highest density interval was found by means of the **hdi** function in the **HDInterval** package for **R** (Meredith & Kruschke, 2018). The highest density interval is a continuous range of values in a sample, [*x*_l_, *x*_h_], such that the target proportion of observations in the sample is included in the interval (here 95%), and the empirical density for all values within the range is larger than for any points outside the range.

### 2.4 Data subsets

Subsets of data on yield and nutrient concentrations for field zones were analysed. The first subsets were divided by crop (feed wheat or milling wheat) and harvest year (2015, 2016 or 2017) and included data on P, K and Mg. We then examined the available data on soil P in greater detail with a view to seeing whether distinct boundary responses could be modelled over soils contrasting with respect to properties measured in the AgSpace Agriculture Ltd. soil zone characterization. Specifically, we examined the boundary responses for subsets of the data defined initially by intervals of (topsoil) pH. The intervals were pH ≤ 7, 7 < pH ≤ 7.5, 7.5 < pH ≤ 8 and pH > 8. For these analyses we combined the milling and feed‐wheat yields together. Although there might be differences in yields of the two crops due to management (e.g., different nitrogen applications), the boundary line for the combined set represents the P response when such effects are not limiting and so allows us to identify target P concentrations over rotations with both milling and feed crops. It also ensured that there were adequate data in each season and subset. We then undertook a similar analysis in which the boundary responses to soil P were modelled for combined feed and milling‐wheat yields over soils of different depth intervals, as recorded by AgSpace Agriculture Ltd.: shallow (depth < 30 cm), medium (30–50 cm) and deep (>50 cm).

## 3 RESULTS

### 3.1 All nutrients

Summary statistics for the datasets are provided in Appendix S1 (Tables S1 and S2). As noted above, it was decided to transform all nutrient concentrations to natural logarithms for the BLA.

Table 1 shows the negative log‐likelihoods, values of AIC (A) and the AIC weights (*w*_*i*_) for all models fitted to the milling and feed‐wheat yield datasets for each season and nutrient. Recall that the boundary line model was selected only when the Akaike weight for that model was *w*_*i*_ ≥ 0.5. On this basis the boundary line model was selected for Mg and both feed and milling wheat in the 2016 and 2017 harvest seasons. The boundary line model was selected for K in all three seasons for milling wheat and for the 2015 season in the case of feed wheat. The boundary line model was selected for P in all three seasons for milling wheat and for all but the 2017 yields in the case of feed wheat.

**Table 1:** Minimized negative log‐likelihoods, AIC (A) and AIC weights (*w*_*i*_) for fitted models for all three nutrients in each crop

Year/crop	Model*	Mg			K			P		
		*ℓ*	A	*w*_*i*_	*ℓ*	A	*w*_*i*_	*ℓ*	A	*w*_*i*_
2015 Milling	BL	8,118.8	16,253.6	0.484	6,936.1	13,888.2	0.966	7,250.9	14,517.8	0.522
	MVN	8,122.1	16,254.1	0.377	6,942.8	13,895.5	0.025	7,254.3	14,518.6	0.350
	MVN_c_	8,122.1	16,256.1	0.139	6,942.8	13,897.5	0.009	7,254.3	14,520.6	0.129
2016 Milling	BL	7,485.1	14,986.2	0.782	6,208.7	12,433.4	0.589	7,502.6	15,021.2	0.532
	MVN	7,491.0	14,992.0	0.043	6,214.1	12,438.2	0.053	7,506.9	15,023.8	0.145
	MVN_c_	7,488.6	14,989.2	0.175	6,211.2	12,434.4	0.357	7,505.1	15,022.2	0.323
2017 Milling	BL	8,161.5	16,339.0	1.000	7,353.8	14,723.6	0.982	8,112.8	16,241.6	0.978
	MVN	8,174.5	16,359.0	0.000	7,361.1	14,732.2	0.013	8,121.4	16,252.8	0.004
	MVN_c_	8,171.4	16,354.8	0.000	7,361.1	14,734.2	0.005	8,118.8	16,249.6	0.018
2015 Feed	BL	12,418.3	24,852.6	0.354	10,090.1	20,196.2	0.550	11,036.6	22,089.1	0.721
	MVN	12,422.7	24,855.4	0.000	10,094.5	20,199.0	0.000	11,121.3	22,252.6	0.000
	MVN_c_	12,419.7	24,851.4	0.646	10,092.3	20,196.6	0.450	11,039.5	22,091.0	0.279
2016 Feed	BL	8,409.4	16,834.8	0.998	7,309.6	14,635.2	0.213	8,124.1	16,264.2	0.996
	MVN	8,424.1	16,858.2	0.000	7,316.0	14,642.0	0.007	8,138.6	16,287.2	0.000
	MVN_c_	8,417.6	16,847.2	0.002	7,310.3	14,632.6	0.780	8,131.7	16,275.4	0.004
2017 Feed	BL	5,175.4	10,366.8	1.000	4,250.6	8,517.2	0.072	4,705.9	9,427.8	0.124
	MVN	5,197.8	10,405.6	0.000	4,251.9	8,513.8	0.395	4,707.8	9,425.6	0.373
	MVN_c_	5,196.4	10,404.8	0.000	4,250.6	8,513.2	0.533	4,706.5	9,425.0	0.503

* BL, bounded linear boundary model; MVN, multivariate normal model; MVN_c_, multivariate normal model with constant censor for yield.

Figure 1 shows 2017 milling‐wheat data and boundary responses to all nutrients. Note that the plots are shown both on the log‐scale for nutrient concentrations and on the original units, the latter plots including the ranges for the RB209 nutrient indices. Plots for the other datasets are in Appendix S1 (Figures S1–S14). The estimated parameters for the boundary line models, where selected, and their standard errors are presented in Table 2.

**Figure 1:**
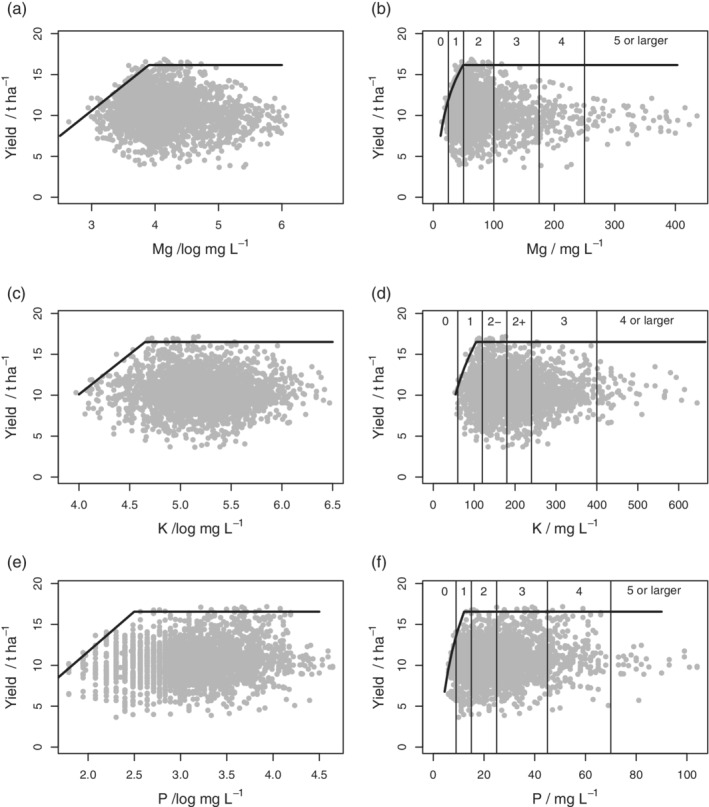
Yield data and soil nutrient concentrations for milling wheat in 2017 with fitted boundary line model. The abscissa of the plot is (a) log‐transformed soil Mg concentration; (b) soil Mg concentration in original units with RB209 Index boundaries shown; (c) log‐transformed soil K concentration; (d) soil K concentration in original units with RB209 Index boundaries shown; (e) log‐transformed soil P concentration; (f) soil P concentration in original units with RB209 Index boundaries shown

**Table 2:** Estimates of boundary line model parameters and their standard errors for all three nutrients in each crop where *w*_BL_ ≥ 0.5

		Mg(log)		K(log)		P(log)	
Crop/year	Parameter	Estimate	Standard error	Estimate	Standard error	Estimate	Standard error
2015 Milling	*β*_2_			16.65	0.38	16.96	0.34
	*β*_0_			−7.80	0.44	4.87	0.85
	*β*_1_			4.82	0.70	4.37	0.95
2016 Milling	*β*_2_	13.64	0.19	13.41	0.17	13.81	0.23
	*β*_0_	−21.05	0.43	−2.72	0.57	5.02	0.36
	*β*_1_	10.15	1.47	3.29	0.90	3.19	0.57
2017 Milling	*β*_2_	16.16	0.26	16.51	0.28	16.56	0.11
	*β*_0_	−7.92	0.38	−29.22	0.39	−7.93	0.35
	*β*_1_	6.17	0.46	9.83	1.53	9.80	1.47
2015 Feed	*β*_2_			16.01	0.45	13.69	0.09
	*β*_0_			−19.37	1.47	6.16	1.0
	*β*_1_			7.68	2.75	2.77	0.41
2016 Feed	*β*_2_	13.43	0.17			13.50	0.87
	*β*_0_	−14.30	0.34			4.95	0.12
	*β*_1_	7.91	0.67			3.08	0.05
2017 Feed	*β*_2_	15.37	0.36				
	*β*_0_	−7.35	0.45				
	*β*_1_	5.62	0.69				

These results show critical concentrations of all nutrients, *x*_crit_, as defined in Equation (12). In the case of Mg, the critical concentration was at the Index 0/1 boundary (milling wheat, Figure S4) and in the Index 1 range (feed wheat, Figure S5) in 2016. In 2017 it was at the Index 1/2 boundary for both feed and milling wheat (Figure 1b, Figure S14). For K the critical concentration was in the index −2 range or near the 1/−2 boundary (e.g., Figure 1d). For P the critical concentration was at the 1/2 boundary in four out of the five cases where the boundary line model was selected (Figures S3, S6, S11, S13); in the fifth case it was within the Index 1 interval (Figure 1f).

### 3.2 P subsets

Table 3 shows the negative log‐likelihoods, values of AIC (A) and the AIC weights (*w*_*i*_) for all models on data subsets defined by intervals of soil pH. Note that the boundary line model was selected on the criterion *w*_*i*_ ≥ 0.5 for pooled datasets in all years. When the subsets are considered, the boundary line model was selected on this criterion in 2015 (two subsets with pH > 7.5), in 2016 (all subsets apart from 7.5 < pH ≤ 8) and in 2017 (subset with pH ≤7).

**Table 3:** Models for pooled wheat yield data and soil P, including data subsets defined by pH intervals

Subset	Model*	Year								
		2015			2016			2017		
		*ℓ*	A	*w*_*i*_	*ℓ*	A	*w*_*i*_	*ℓ*	A	*w*_*i*_
All data	BL	18,032.2	36,080.3	0.879	15,721.6	31,459.1	1.000	12,817.8	25,651.7	0.935
	MVN	18,041.9	36,093.9	0.001	15,737.5	31,485	0.000	12,823.8	25,657.6	0.048
	MVN_c_	18,036.2	36,084.9	0.120	15,734.9	31,481.8	0.000	12,823.8	25,659.6	0.018
pH ≤7	BL	3,961.9	7,939.8	0.335	3,538.8	7,093.7	0.946	2,788.1	5,592.3	0.997
	MVN	3,965.0	7,940.0	0.312	3,545	7,100	0.04	2,797.2	5,604.3	0.002
	MVN_c_	3,963.9	7,939.7	0.352	3,545	7,102	0.015	2,797.2	5,606.3	0.001
7 < pH ≤ 7.5	BL	3,035.5	6,086.9	0.05	3,057.6	6,131.3	0.941	2,137.3	4,290.6	0.108
	MVN	3,036.0	6,082.1	0.576	3,065.8	6,141.6	0.005	2,138.5	4,287.0	0.654
	MVN_c_	3,035.5	6,082.9	0.373	3,062.5	6,137	0.054	2,138.5	4,289.0	0.238
7.5 < pH ≤ 8	BL	5,539.4	11,094.9	0.997	4,112.9	8,241.8	0.312	3,420.7	6,857.3	0.354
	MVN	5,550.0	11,110.1	0.001	4,116.4	8,242.6	0.206	3,423.4	6,856.7	0.473
	MVN_c_	5,547.3	11,106.7	0.003	4,114.5	8,240.9	0.482	3,423.4	6,858.7	0.174
pH > 8	BL	5,649.9	11,315.8	0.631	4,902.7	9,821.4	0.874	4,203.5	8,423	0.183
	MVN	5,654.4	11,318.8	0.141	4,908.4	9,826.8	0.058	4,205.3	8,420.6	0.598
	MVN_c_	5,652.9	11,317.9	0.229	4,907.3	9,826.5	0.068	4,205.3	8,422.6	0.22

*Note*: Minimized negative log‐likelihoods, AIC (A) and AIC weights (*w*_*i*_) for fitted models.

* BL, bounded linear boundary model; MVN, multivariate normal model; MVN_c_, multivariate normal model with constant censor for yield.

**Figure 2:**
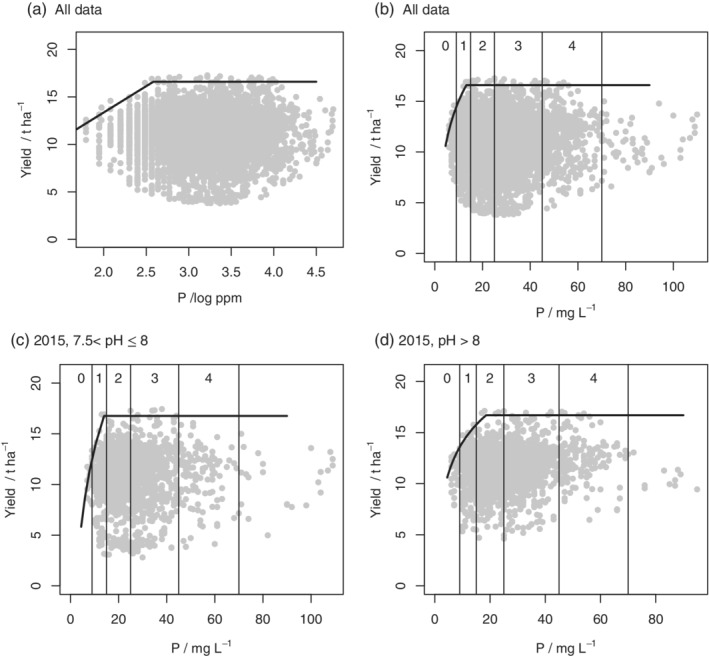
Yield data from 2015 and soil P concentration and fitted boundary model for all wheat data on (a) the log scale or (b) the original scale with index boundaries shown and subsets defined by pH (c) 7.5 < pH ≤8 and (d) pH > 8

**Figure 3:**
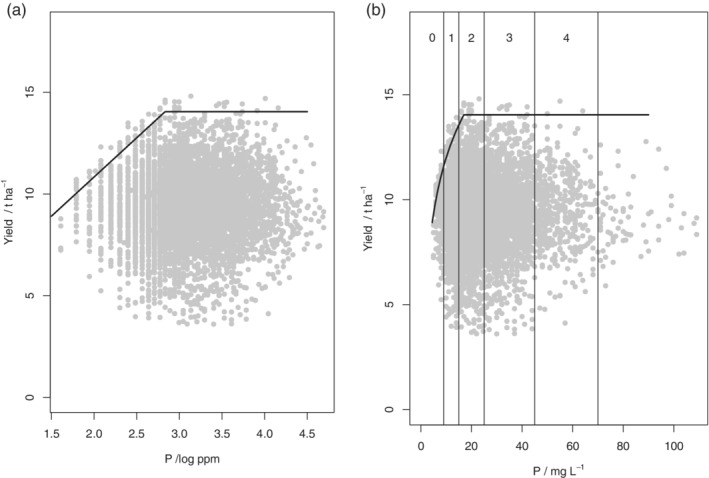
Yield data from 2016 and soil P concentration and fitted boundary model for all wheat data on (a) the log scale or (b) the original scale with index boundaries shown

**Figure 4:**
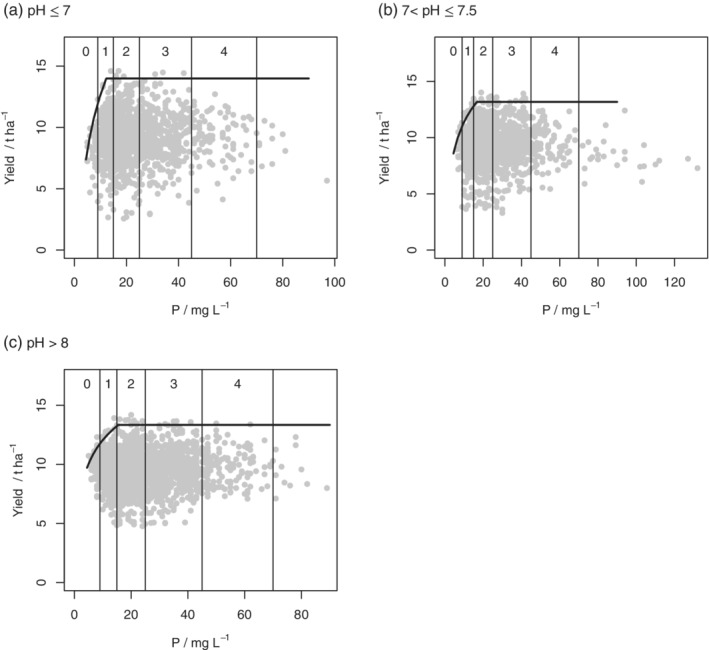
Yield data from 2016 and soil P concentration and fitted boundary model for all wheat data on the original scale with index boundaries shown and subsets defined by pH (a) pH ≤7, (b) 7 < pH ≤7.5 and (c) pH > 8

**Figure 5:**
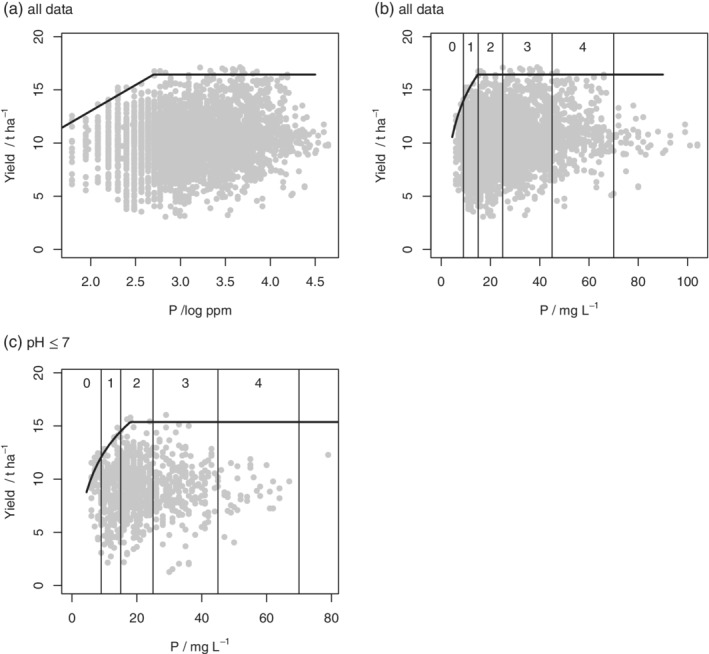
Yield data from 2017 and soil P concentration and fitted boundary model for all wheat data on (a) the log scale or (b) the original scale with index boundaries shown and (c) subset defined by pH, pH ≤7

The parameters of the boundary line model, where selected, and their standard errors are presented in Table 4. Figures 2, 3, 4, 5 show the data and fitted models, again with nutrients plotted on both the log and original scales with RB209 Index ranges indicated in the latter case.

**Table 4:** Models for pooled wheat yield data and soil P, including data subsets defined by pH intervals

Subset	Parameter	Year					
		2015		2016		2017	
		Estimate	Standard error	Estimate	Standard error	Estimate	Standard error
All data	*β*_2_	16.6	0.18	14.04	0.13	16.44	0.14
	*β*_0_	2.28	2.5	3.13	0.14	3.22	1.65
	*β*_1_	5.54	1.26	3.85	0.03	4.9	0.71
pH ≤7	*β*_2_			13.99	0.33	15.37	0.64
	*β*_0_			−2.46	1.73	1.65	1.07
	*β*_1_			6.56	0.92	4.74	0.47
7 < pH ≤7.5	*β*_2_			13.17	0.31		
	*β*_0_			3.35	1.49		
	*β*_1_			3.49	0.62		
7.5 < pH ≤8	*β*_2_	16.76	0.27	·			
	*β*_0_	−8.57	1.69	·			
	*β*_1_	9.6	0.72	·			
pH > 8	*β*_2_	16.69	0.29	13.48	0.35		
	*β*_0_	4.16	2.33	5.05	1.26		
	*β*_1_	4.29	1.09	3.07	0.6		

*Note*: Estimates of boundary line model parameters and their standard errors where *w*_BL_ ≥ 0.5.

**Figure 6:**
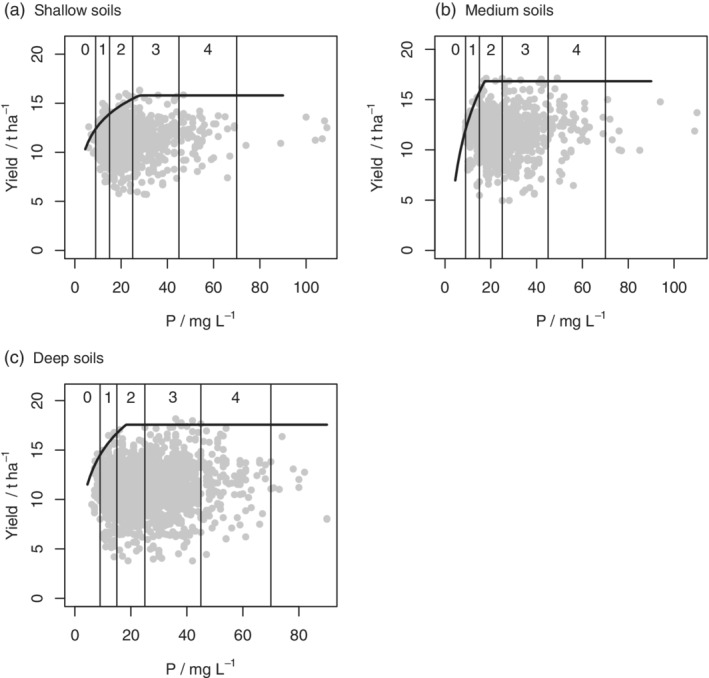
Yield data from 2015 and soil P concentration and fitted boundary model for data subsets defined by depth: (a) shallow (<30 cm), (b) medium (30–50 cm) and (c) deep (>50 cm)

**Figure 7:**
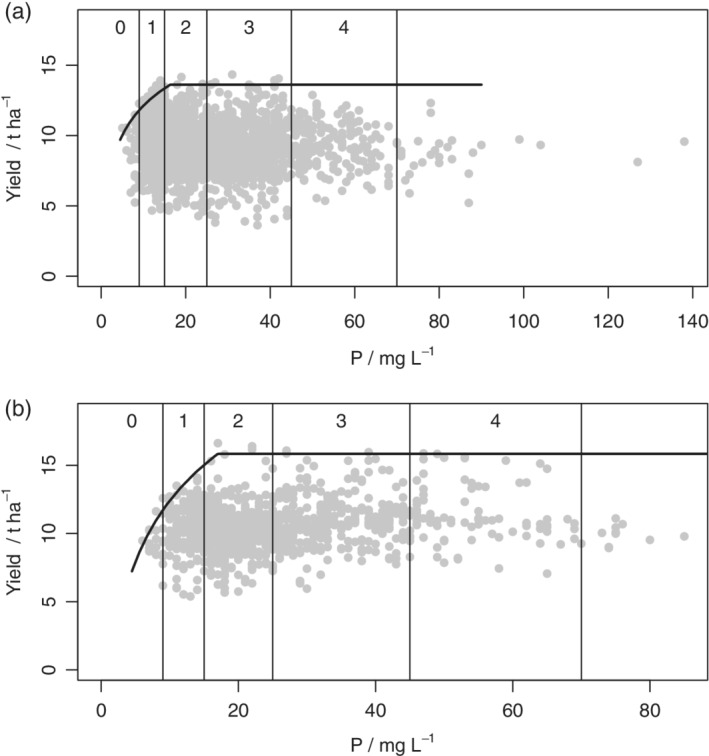
(a) Yield data and soil P concentration with fitted boundary model for (a) all wheat data in 2016 over deep soils (>50 cm) and (b) all wheat data in 2017 over shallow soils (<30 cm)

Table 5 shows the negative log‐likelihoods, values of AIC (A) and the AIC weights (*w*_*i*_) for all models on data subsets defined by intervals of soil depth. The boundary line model was selected for the datasets for 2015 (all depth intervals), 2016 (deep soil only) and 2017 (shallow soil only). Model parameters and standard errors for the selected boundary line models are presented in Table 6 and plots of the data and fitted models are in Figures 6 and 7.

**Table 5:** Models for pooled wheat yield data and soil P, including data subsets defined by depth intervals. Minimized negative log‐likelihoods, AIC (A) and AIC weights (*w*_*i*_) for fitted models

Subset	Model*	Year								
		2015			2016			2017		
		*ℓ*	A	*w*_*i*_	*ℓ*	A	*w*_*i*_	*ℓ*	A	*w*_*i*_
Shallow	BL	2,711.1	5,438.1	0.582	2,365.8	4,747.5	0.223	2,500.4	5,016.8	0.634
(< 30 cm)	MVN	2,715.0	5,439.9	0.237	2,368.0	4,746.0	0.467	2,504.5	5,018.9	0.226
	MVN_c_	2,714.2	5,440.5	0.182	2,367.4	4,746.9	0.310	2,503.9	5,019.9	0.139
Medium	BL	2,318.6	4,653.1	0.965	1958.5	3,933.0	0.055	2078.8	4,173.6	0.130
(30–50 cm)	MVN	2,325.7	4,661.3	0.016	1959.0	3,927.9	0.681	2080.3	4,170.6	0.584
	MVN_c_	2,324.5	4,661.0	0.019	1958.9	3,929.8	0.265	2080.0	4,172.0	0.286
Deep	BL	6,115.4	12,246.7	0.578	5,131.6	10,279.2	0.84	5,426.9	10,870.0	0.111
(>50 cm)	MVN	6,119.4	12,248.9	0.199	5,429.0	10,284.9	0.049	5,429.0	10,868.1	0.285
	MVN_c_	6,118.3	12,248.6	0.222	5,427.3	10,283.3	0.111	5,427.3	10,866.6	0.604

* BL, bounded linear boundary model; MVN, multivariate normal model; MVN_c_, multivariate normal model with constant censor for yield.

**Table 6:** Models for pooled wheat yield data and soil P, including data subsets defined by depth intervals

Subset	Parameter	Year					
		2015		2016		2017	
		Estimate	Standard error	Estimate	Standard error	Estimate	Standard error
Shallow	*β*_2_	15.79	0.21			15.84	0.27
	*β*_0_	5.81	0.22			−2.52	0.28
	*β*_1_	2.99	0.03			6.48	0.04
Medium	*β*_2_	16.83	0.36				
	*β*_0_	−3.97	3.09				
	*β*_1_	7.29	1.34				
Deep	*β*_2_	17.56	0.39	13.62	0.37		
	*β*_0_	5.06	2.17	5.15	1.42		
	*β*_1_	4.3	0.95	3.03	0.61		

*Note*: Estimates of boundary line model parameters and their standard errors where *w*_BL_ ≥ 0.5.

The values of *x*_crit_, which define the inflexion points for the soil P boundary lines, are presented in Table 7 along with the bounds for their 95% confidence intervals. Also presented are the probabilities that the inflexion point lies in each RB209 Index range for soil P. In 2015 and 2016 there is evidence that the boundary P response was somewhere in Index 2 for soils of pH > 8 (Figures 2d and 4c), but the critical value was smaller for soils with pH <7 (Figure 4a). In 2016 the inflexion point for soils with pH ≤7 was most likely in Index 1 (Figure 4a), although in 2017 it was in Index 2 (Figure 5c).

**Table 7:** Inflexion points with confidence intervals and probability that the inflexion point lies in each P index range for all datasets and subsets defined by pH or by depth where *w*_BL_ ≥ 0.5

		Inflexion point/mgL^−1^			Index probability†					
Year	Dataset	Estimate	Upper*	Lower*	0	1	2	3	4	5
2015	All data	13.0	10.1	19.6	0	0.76	0.23	0.01	0	0
	7.5 < pH ≤	14.0	13.3	14.8	0	0.99	0.01	0	0	0
	pH > 8	18.6	12.1	35	0	0.12	0.71	0.14	0.02	0
	Shallow	28.1	26.7	29.4	0	0	0	1	0	0
	Medium	17.3	14.0	22.8	0	0.06	0.92	0.02	0	0
	Deep	18.3	12.9	28.5	0	0.09	0.81	0.09	0	0
2016	All	17.0	16.4	17.6	0	0	1	0	0	0
	pH ≤ 7	12.3	9.8	15.7	0	0.93	0.07	0	0	0
	7 < pH ≤ 7.5	16.7	13	21.9	0	0.17	0.82	0.01	0	0
	pH > 8	15.6	10.7	24.0	0	0.42	0.54	0.03	0	0
	Deep	16.4	11.5	24.0	0	0.3	0.67	0.03	0	0
2017	All	14.9	12.6	17.9	0	0.53	0.47	0	0	0
	pH ≤7	18.0	13.0	24.3	0	0.1	0.87	0.03	0	0
	Shallow	17.0	16.6	17.5	0	0	1	0	0	0

* Upper and lower bounds of the 95% confidence intervals (highest density interval).

† Probability that the inflexion point falls in each index interval.

In the 2015 data, partitioned by soil depth, critical P concentrations appear to be larger for shallow soils (into Index 3) 2017 (Figure 6a). In 2016 the critical value for deep soils was close to the 1/2 boundary (Figure 7a) and in 2017 the critical value for shallow soils was in Index 2 (Figure 7b). As might be expected, Table S3 (2015 data) shows that shallow soils have larger pH than medium or deep ones, so these two subdivisions of the data are not independent. Figure S15 shows the yield and soil P data (2015) for shallow soils, with soils of very large pH (>8) distinguished from the rest. It is notable (Figure S15 and Table S3) that the soils at sites close to the boundary line and below the inflexion point are generally of larger pH than soils below the boundary. This suggests that P limitations are particularly likely for the shallow soils of largest pH.

## 4 DISCUSSION

### 4.1 General observations

It is interesting that the boundary line model is commonly preferable to the multivariate normal distribution or multivariate normal with a constant censor to represent the joint variation of soil nutrient concentrations and crop yield. There are some differences in model behaviour between seasons, although the boundary line model is favoured over the alternatives in most cases considered. Variation in behaviour between the seasons is to be expected as general biophysical limitations vary (e.g., weather, pest and disease severity) as well as other inputs (e.g., nitrogen fertilizer due to variations in both cost and the commodity price). These differences may affect the maximum yield achievable in the season, and so *x*_crit_, and may also interact with potentially limiting factors. They may also result in limited or no expression of the boundary in the dataset for a particular season, in the sense of Condition (4). It is, therefore, necessary to build an evidence base for boundary line models over multiple seasons. This study is a significant start. It should also be borne in mind that this study is based on data from farms investing significantly in data to improve the management of their crop. Such farms might be expected to be well managed in other respects (e.g., with respect to soil drainage, acidity and physical limitations such as compaction), and although this would not be expected to bias the estimate of the boundary line, the overall distribution of observations relative to the boundary might not be representative of farmed UK soils as a whole.

We note that the procedure used here to analyse large datasets from across the UK is based on a statistical model and advances a hypothesis that can be tested against alternatives to quantify the strength of evidence to support it. We think this is a strength of the approach by comparison to machine learning methods, which search for patterns and correlations within large datasets rather than testing hypotheses in a model‐based framework. Machine learning methods can be used to build complex models that relate crop yields to multiple attributes of a field or soil zone. However, the complexity of the models means that it is difficult to interpret the causes of yield variation. Where this is the aim, we would encourage, in general, the development of hypothesis‐driven approaches to big data based on statistical modelling principles.

### 4.2 Overall models for all nutrients

It is informative to compare the overall results, without subsetting on soil properties, with RB209 recommendations (AHDB, 2017). For K, RB209 recommends that soils are maintained at target index −2. That is consistent with the results obtained here, where the value of *x*_crit_ for K was near the Index 1/−2 boundary or in Index −2. Similarly for P, the RB209 target Index is 2, which is consistent with the overall results presented in this paper.

It should be noted that these results do not simply confirm RB209. Those recommendations are based on experiments designed to estimate mean crop responses at the field scale or indeed at the farm scale. Non‐linear responses of soil–plant systems do not, in general, remain invariant with change of scale, an issue that has been specifically addressed by Kachanoski and Fairchild (1995) in respect of fertilizer recommendations at scales around the whole field. The robustness of the RB209 recommendations for sub‐field zones, at least for P and K, is therefore of some interest and suggests that they could provide a basis for improved nutrient management at the within‐field scale with precision agriculture technology.

For wheat crops RB209 recommends that Mg is added in fertilizer to soils in Index 0, but the results presented in this paper suggest that, in some seasons, Mg concentrations in the Index 1 range can be limiting. Note that Index 2 is the target for grass in the RB209 system, although this is determined by considerations of the requirements for grazing livestock as well as the growth of the crop. Lark, Ander, and Broadley (2019) present a map of the probability that soil Mg is below Index 2 for soils in agricultural use across England and Wales. This shows that if soil Mg status below Index 2 is potentially limiting on wheat yield, then this could be an issue in parts of the south, north‐east and east of England, particularly in soils over chalk.

### 4.3 Subsets

Within‐season comparisons suggest that critical *P* values may be larger on soils of larger pH (into index 2). The maximum yield penalty for soils at Index 0 rather than at the threshold can be of the order of 4 t ha^−1^. Maximum yield penalty means the potential yield response to an increase in the P status to the threshold, on the assumption that P remains limiting and some other factor does not become so.

These results also provide evidence that shallow soils may be more susceptible than deeper ones to P limitation with the inflexion point in the boundary response at *x*_crit_ approaching or in Index 3. Figure S15 and Table S3 show that the effects of soil depth and of pH are related, with the mean pH much larger in shallow soils than in deeper ones, and with the soils of largest pH dominating in the vicinity of the boundary response to P for shallow soils. It is not unexpected that soil P availability is reduced in calcareous conditions (Delgado & Torrent, 2000) and this effect could be increased in shallow soil where the rooting depth is also limited. The results presented here suggest that this has implications for the target P concentrations in the soil, or other management practices, to avoid limiting effects on crop yield.

## 5 CONCLUSIONS

To conclude, we have shown in several instances that there is strong statistical evidence for a boundary line model over alternatives to represent the joint variation of soil nutrient status and crop yield, and that this model provides a basis for identifying target nutrient indices to support fertilizer practice. The results obtained here show that, for P and K, the overall boundary line response obtained for within‐field zones is consistent with the set of Index values in the RB209 recommendation system used for field‐ and farm‐scale fertilizer management in the UK. This suggests that RB209 Index values could be used as guidelines for spatially variable management of these nutrients at the within‐field scale. However, the boundary line model provided evidence that recommendations for Mg fertilizer application to winter wheat might not be adequate and that larger target index values should be maintained. This could be based on within‐field soil management zones, such as those defined by AgSpace Agriculture Ltd. We suggest that the boundary line methodology used here could be applied to the evaluation of index values for soil fertility management under other systems.

Examination of boundary line models for P in subsets of the data defined by pH or soil depth intervals, suggested that P advice might be tailored to local soil conditions, with larger target P concentrations specified for shallow soils with large pH to avoid the loss of yield and suboptimal use by the crop of other inputs.

## Supporting information


**Appendix S1** Supporting information.

## Data Availability

The data reported in this paper are commercial and in confidence, and as such are not available to third parties.
