# Supplementary material for: Boundary line models for soil nutrient concentrations and wheat yield in national‐scale datasets
Source: Eur J Soil Sci. 2019 Nov 15;71(3):334–51. doi: 10.1111/ejss.12891 (PMC7318209; doi:10.1111/ejss.12891)
Supplement: Supplementary file 1 — Appendix S1 Supporting information. [file EJSS-71-334-s001.pdf]

Boundary line models for soil nutrient concentrations and wheat yield in national-scale data sets.

R.M. Lark[1], V. Gillingham[2], D. Langton[3] & B.P. Marchant[4].

[1]*School of Biosciences, University of Nottingham, Sutton Bonington, Nottinghamshire LE12 5RD, U.K.,* [2]*AgSpace Agriculture Ltd., Dorcan Business Village, Murdock Road, Swindon, SN3 5HY, U.K.,* [3]*Origin Enterprises, plc, 4–6 Riverwalk, Citywest Business Campus, Dublin 24, Ireland,* [4]*British Geological Survey, Keyworth, Nottinghamshire NG12 5GG, U.K.*

Correspondence: R.M. Lark. E-mail: murray.lark@nottingham.ac.uk

Supplementary Information

Table S1. Milling wheat. Summary statistics of raw and transformed data.

	Yield	Mg		K		P	
	t ha^{-1}	mg kg^{-1}	log mg kg^{-1}	mg kg^{-1}	log mg kg^{-1}	mg kg^{-1}	log mg kg^{-1}
2015							
Mean	11.29	83.2	4.32	201.33	5.23	25.40	3.14
Median	11.43	63.	4.14	185	5.22	23	3.14
Min	2.76	18.0	2.89	55	4.01	5	1.61
Max	19.00	520.0	6.25	760	6.63	5	1.61
Skewness	−0.05	3.1	0.99	1.55	0.08	1.45	0.01
SD	2.22	68.0	0.57	84.57	0.39	11.44	0.43
N^*_{fence}	2699		2677		2654		2664
2016							
Mean	9.28	79.39	4.21	204.33	5.25	27.07	3.17
Median	9.39	61	4.11	190	5.25	23	3.12
Min	0.61	18	2.89	50	3.91	5	1.61
Max	14.53	624	6.44	715	6.57	114	4.74
Skewness	−0.66	3.36	0.91	1.55	0.05	1.36	−0.01
SD	1.70	59.50	0.53	79.82	0.37	14.12	0.50
N^*_{fence}	2931		2875		2843		2895
2017							
Mean	10.44	80.54	4.18	200.38	5.22	25.48	3.11
Median	10.46	60	4.09	187	5.23	22	3.09
Min	2.3	14	2.64	15	2.71	6	1.79
Max	17.55	899	6.80	667	6.50	104	4.64
Skewness	0.02	4.69	1.10	1.28	−0.41	1.52	0.10
SD	2.17	74.91	0.57	80.54	0.40	13.43	0.49
N^*_{fence}	2837		2785		2811		2825

*Number of observations inside the outer fence of the bagplot with yield. Number in Yield column is total sample size.

Table S2. Feed wheat. Summary statistics of raw and transformed data.

	Yield	Mg		K		P	
	t ha^{-1}	mg kg^{-1}	log mg kg^{-1}	mg kg^{-1}	log mg kg^{-1}	mg kg^{-1}	log mg kg^{-1}
2015							
Mean	10.96	92.42	4.26	190.43	5.17	25.61	3.13
Median	11.35	61	4.11	174	5.16	22	3.09
Min	1.72	16	2.77	42	3.74	5	1.61
Max	18.06	1006	6.91	1143	7.04	141	4.95
Skewness	−0.89	3.82	0.99	2.95	0.36	2.44	0.41
SD	2.36	92.5	0.65	87.1	0.39	14.26	0.47
N^*_{fence}	3941		3895		3857		3886
2016							
Mean	9.38	83.63	4.23	190.96	5.17	24.43	3.07
Median	9.44	63	4.14	176	5.17	21	3.04
Min	1.12	16	2.77	49	3.89	4	1.39
Max	16.21	1303	7.17	1178	7.07	138	4.93
Skewness	−0.29	5.82	0.86	3.10	0.33	2.50	0.27
SD	1.90	72.68	0.57	87.56	0.39	14.00	0.49
N^*_{fence}	3061		3008		3011		3023
2017							
Mean	10.22	96.90	4.30	186.45	5.15	24.77	3.09
Median	10.38	62	4.10	172	5.15	21	3.04
Min	1.27	17	2.83	48	3.87	3	1.10
Max	17.02	1459	7.29	1178	7.07	91	4.51
Skewness	−0.33	5.37	1.19	3.03	0.01	1.50	0.15
SD	2.04	111.70	0.65	83.04	0.40	13.20	0.49
N^*_{fence}	1712		1699		1686		1694

*Number of observations inside the outer fence of the bagplot with yield. Number in Yield column is total sample size.

Table S3. Mean and 95% confidence interval for soil pH within each depth class (2015 data). For the shallow soils the mean and confidence interval are also given separately for sites with soil P less than the fitted inflexion point for the boundary model (all wheat yields in the shall soil depth class), and either close to the boundary or more than $2\sigma_0$ units below it.

Subset		Mean pH	95% confidence interval
Shallow	All data	8.07	$[8.05, 8.09]$
Shallow	Soil P below inflexion point		
	Above or within $2\sigma_0$ units of boundary	8.16	$[8.10, 8.22]$
	More than $2\sigma_0$ units below boundary	8.06	$[8.04, 8.08]$
Medium	All data	7.88	$[7.84, 7.92]$
Deep	All data	7.42	$[7.40, 7.44]$

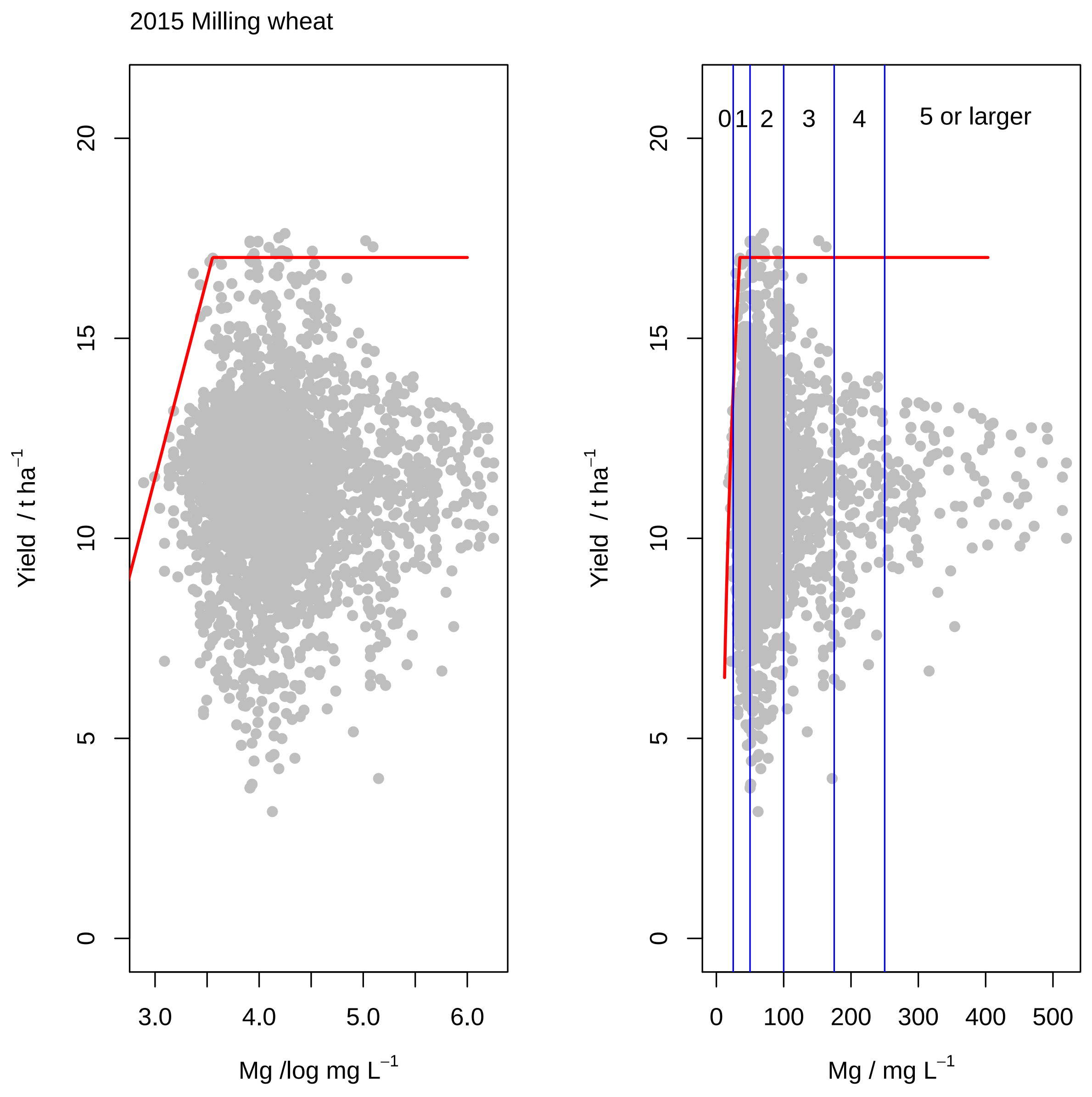

Supplementary Figure 1: Milling wheat, 2015, Mg response

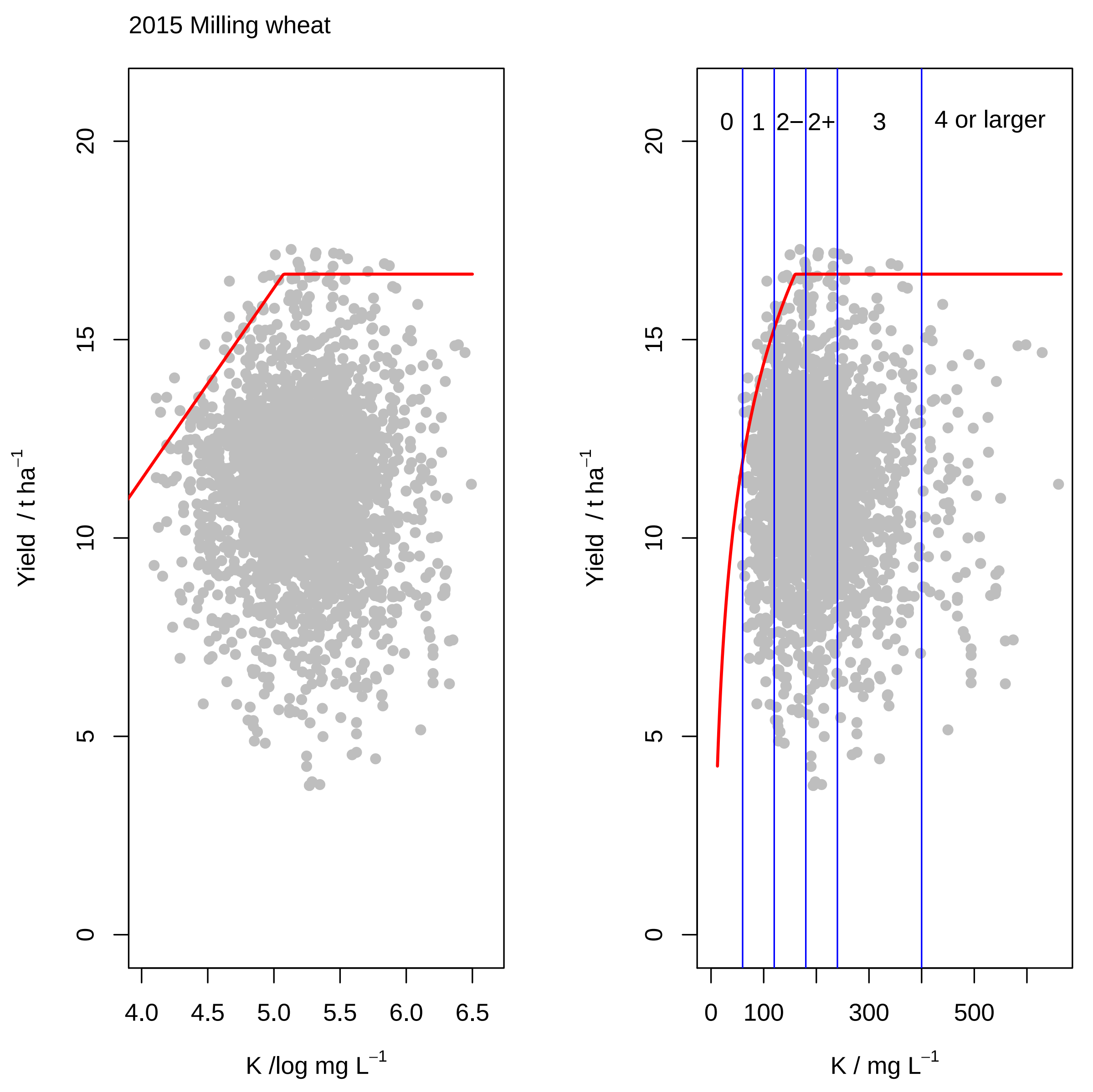

Supplementary Figure 2: Milling wheat, 2015, K response

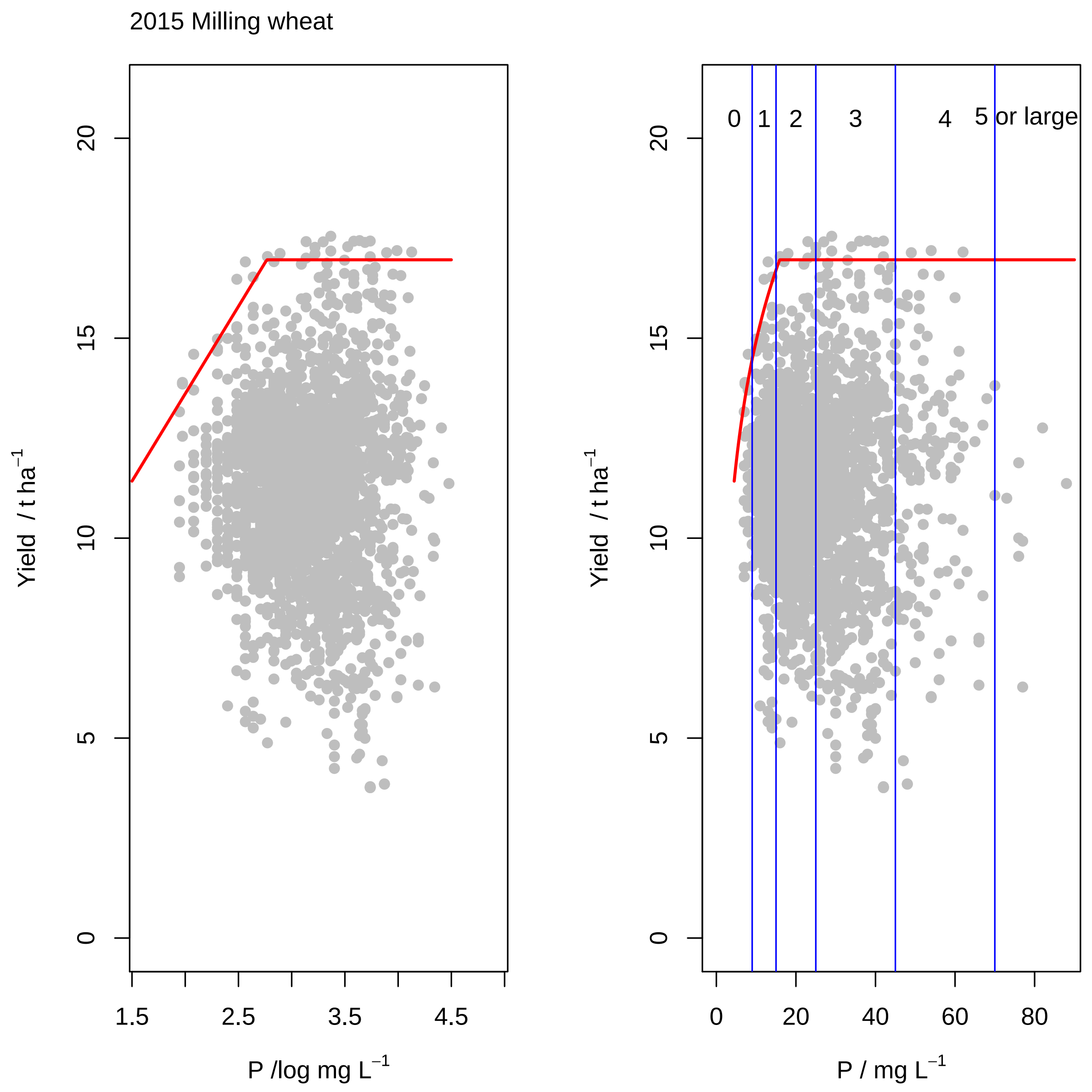

Supplementary Figure 3: Milling wheat, 2015, P response

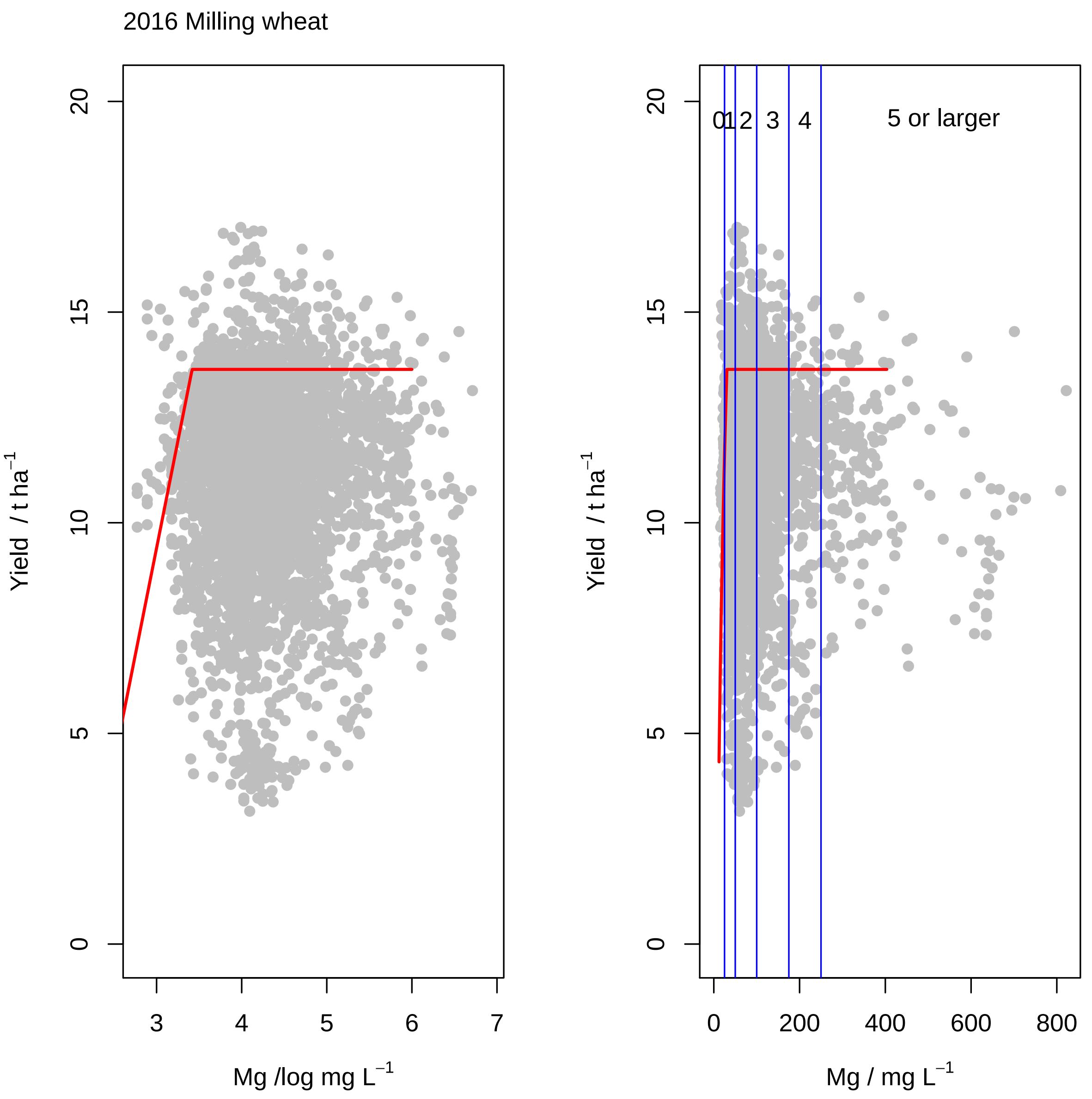

Supplementary Figure 4: Milling wheat, 2016, Mg response

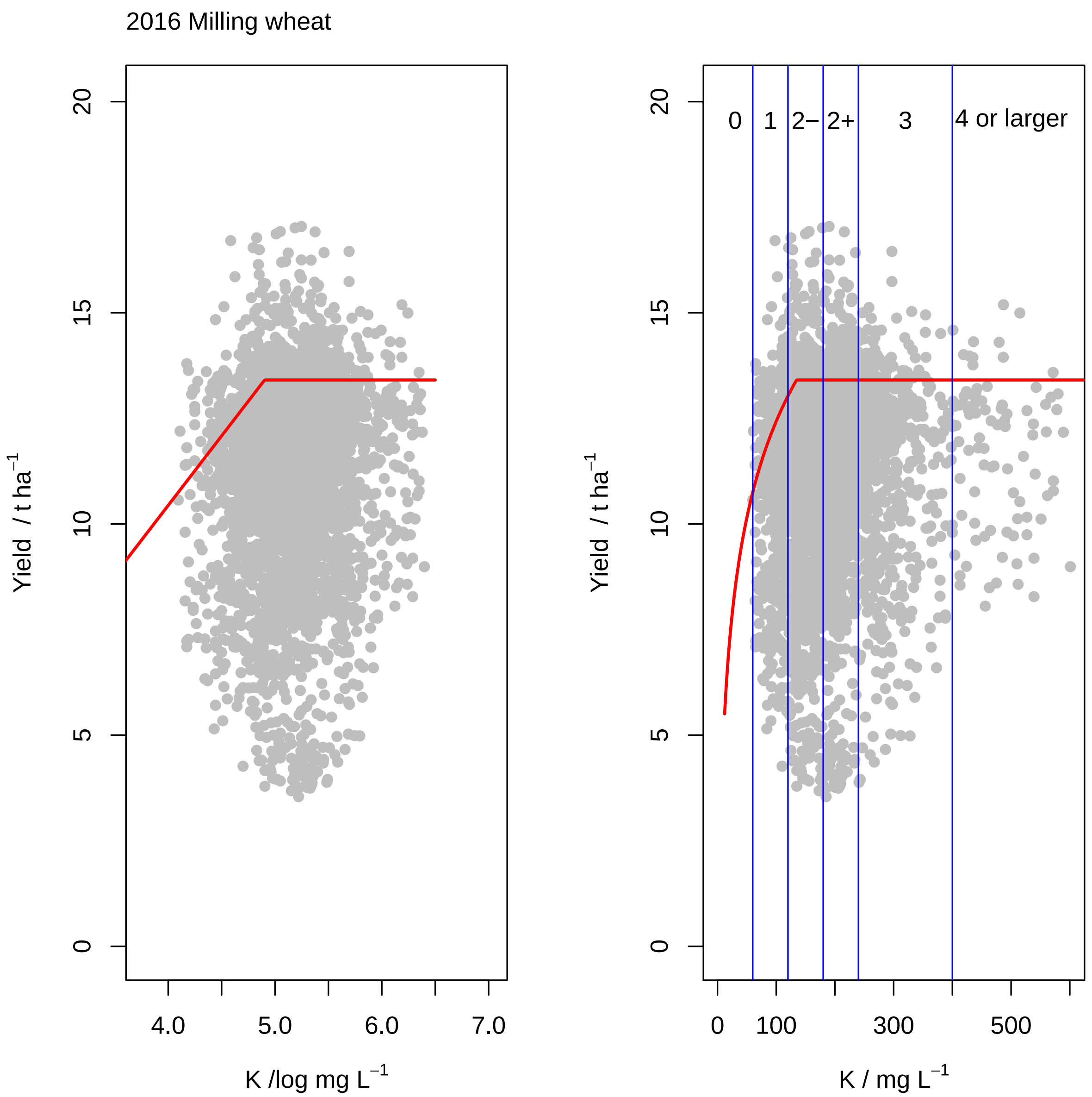

Supplementary Figure 5: Milling wheat, 2016, K response

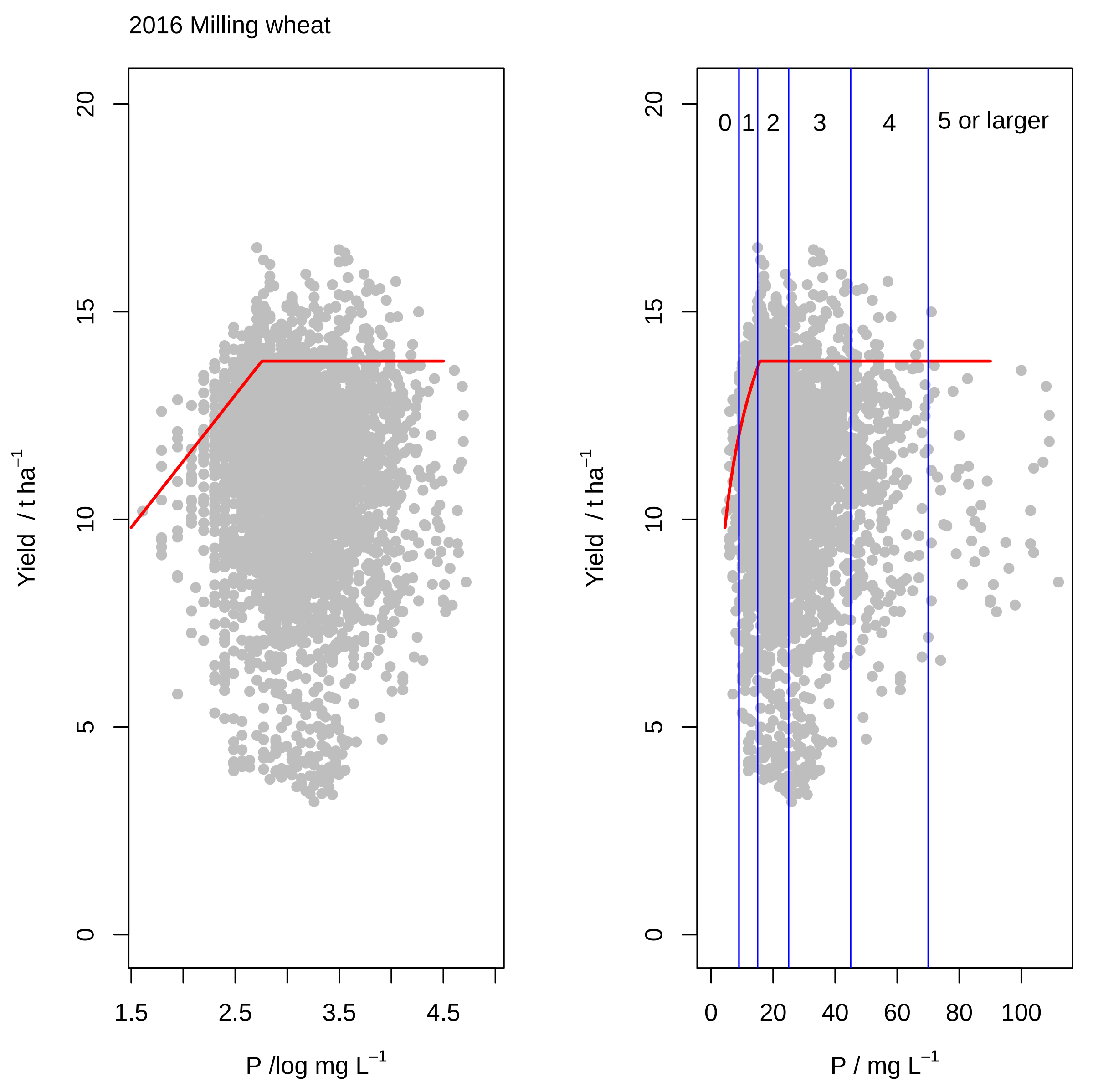

Supplementary Figure 6: Milling wheat, 2016, P response

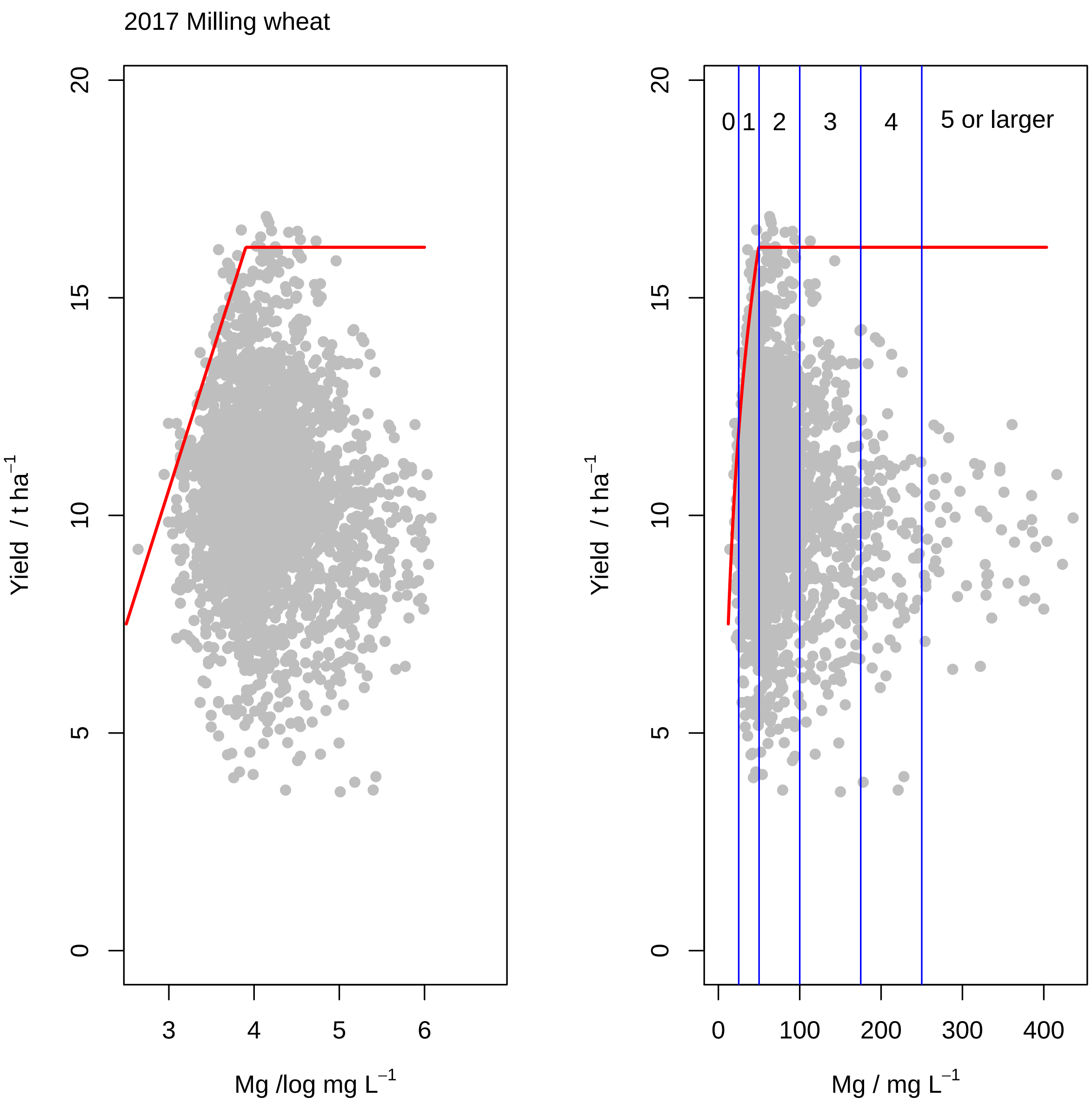

Supplementary Figure 7: Milling wheat, 2017, Mg response

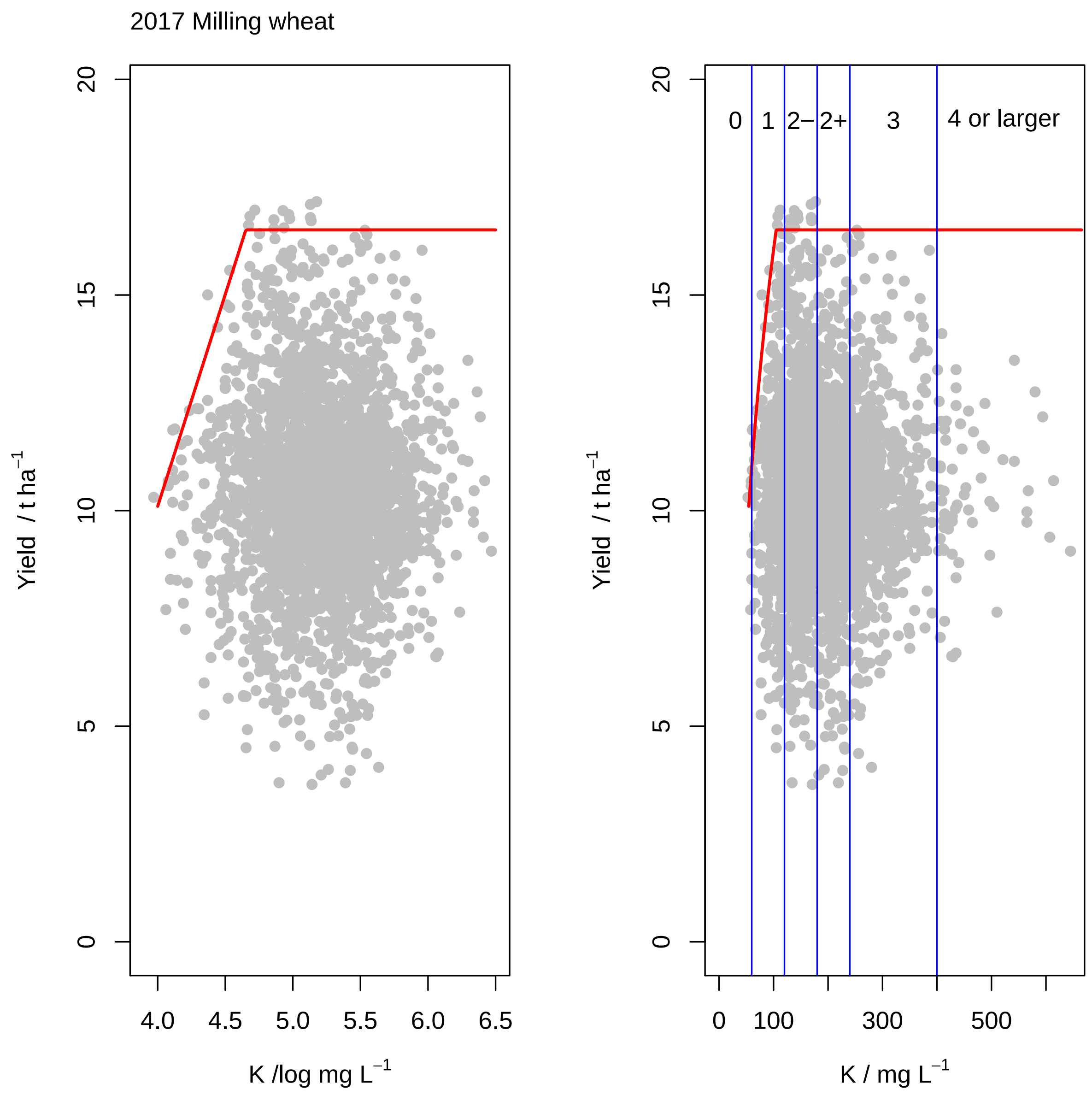

Supplementary Figure 8: Milling wheat, 207, K response

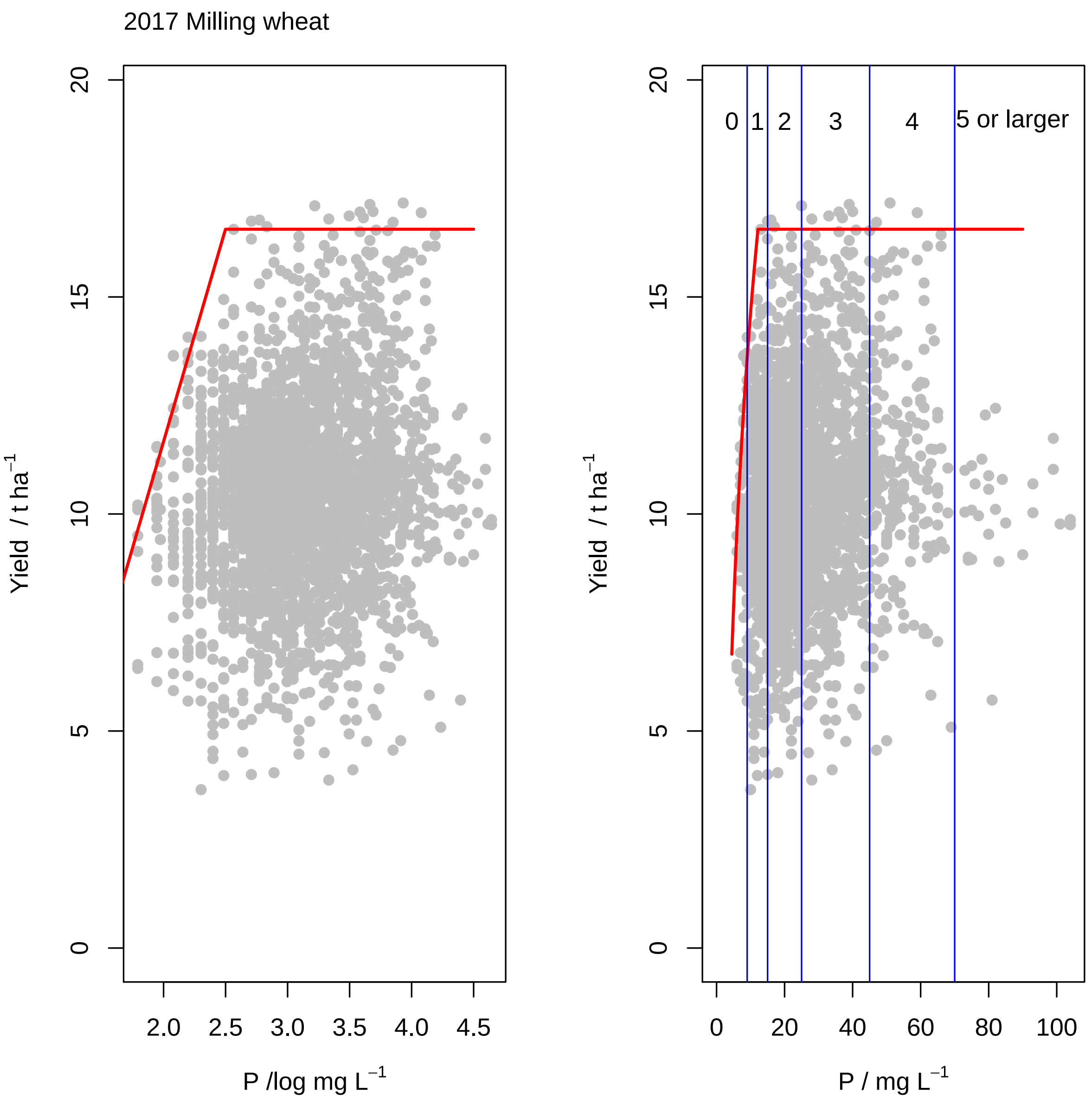

Supplementary Figure 9: Milling wheat, 2017, P response

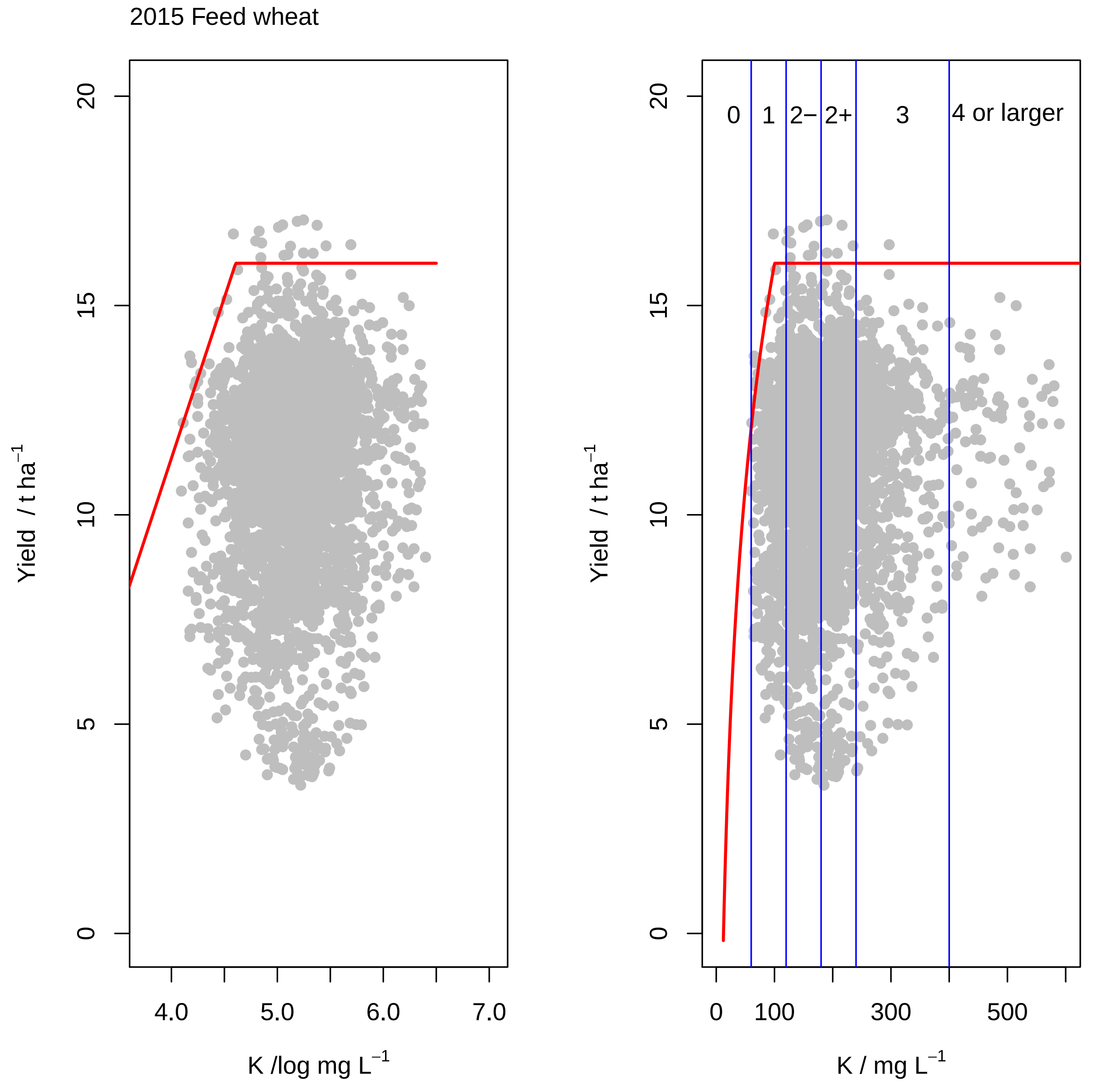

Supplementary Figure 10: Feed wheat, 2015, K response

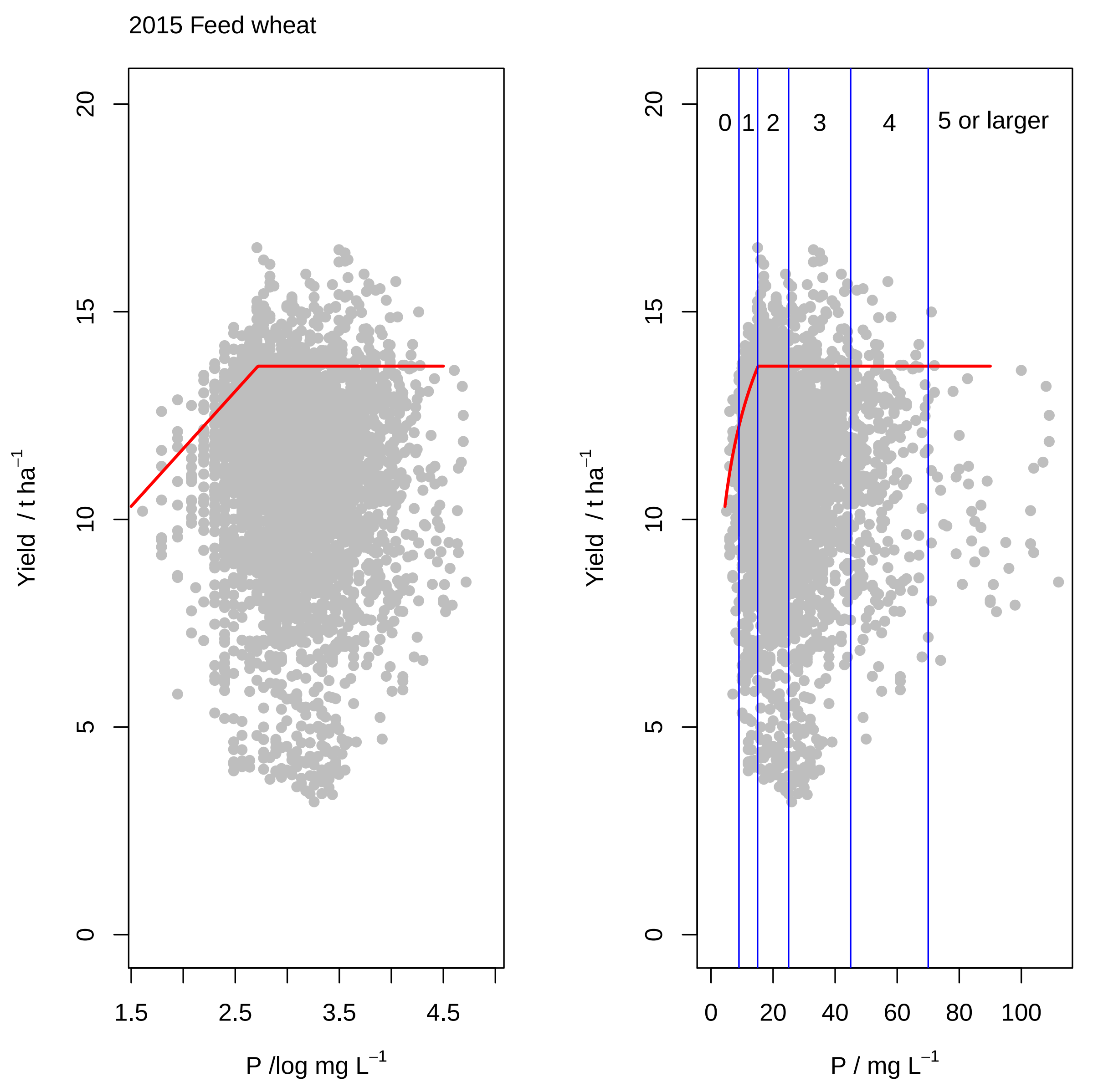

Supplementary Figure 11: Feed wheat, 2015, P response

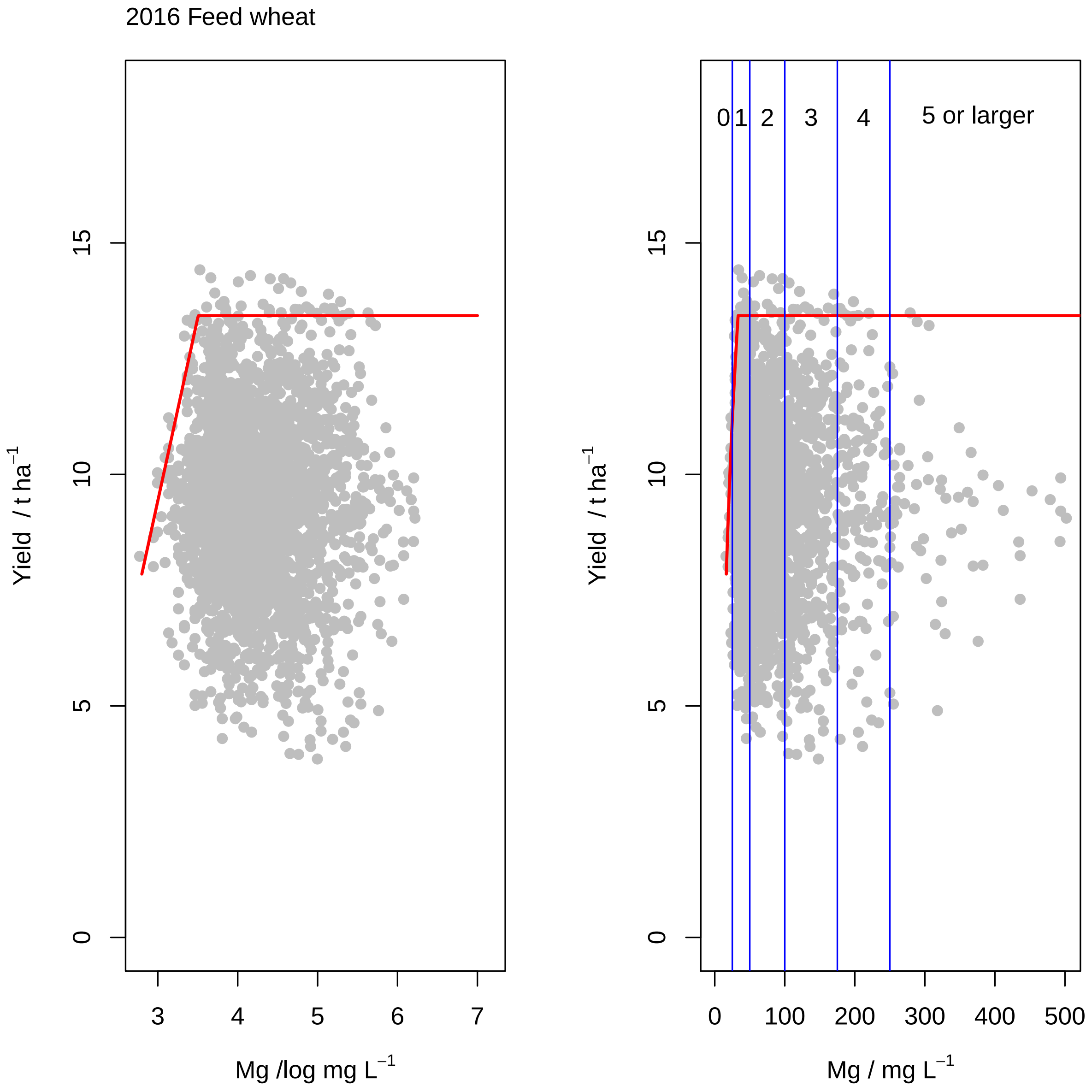

Supplementary Figure 12: Feed wheat, 2016, Mg response

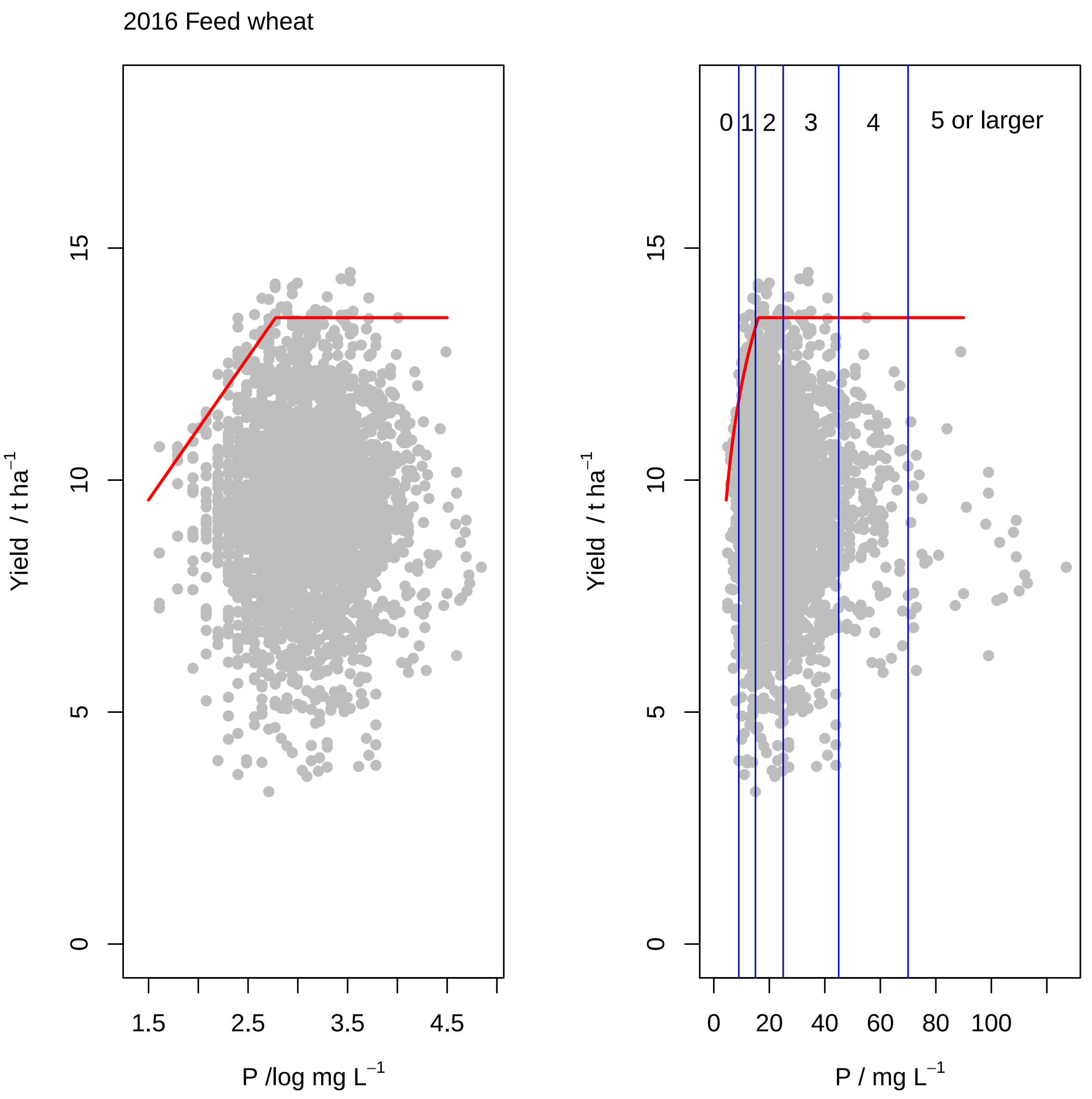

Supplementary Figure 13: Feed wheat, 2016, P response

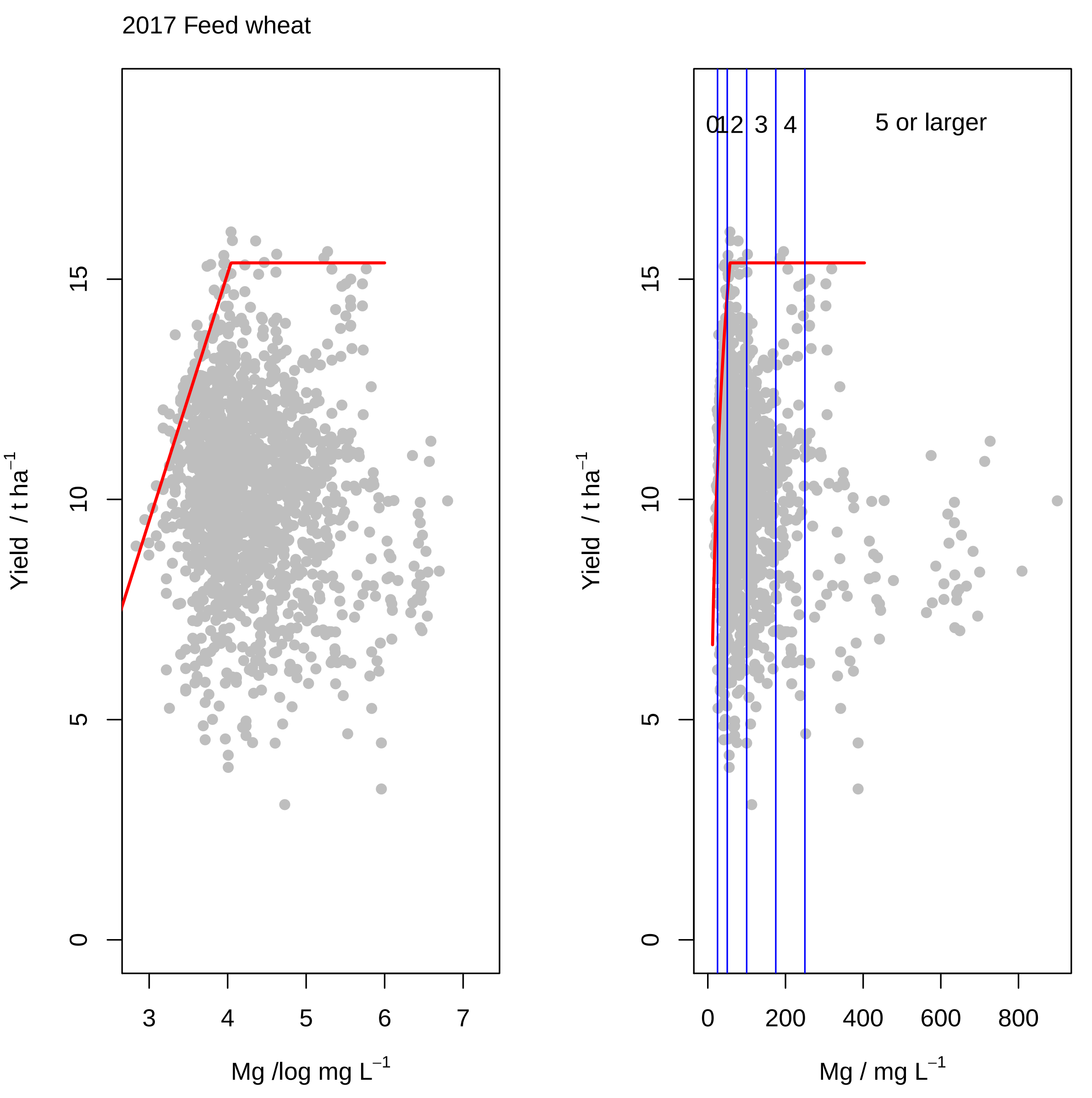

Supplementary Figure 14: Feed wheat, 2017, Mg response

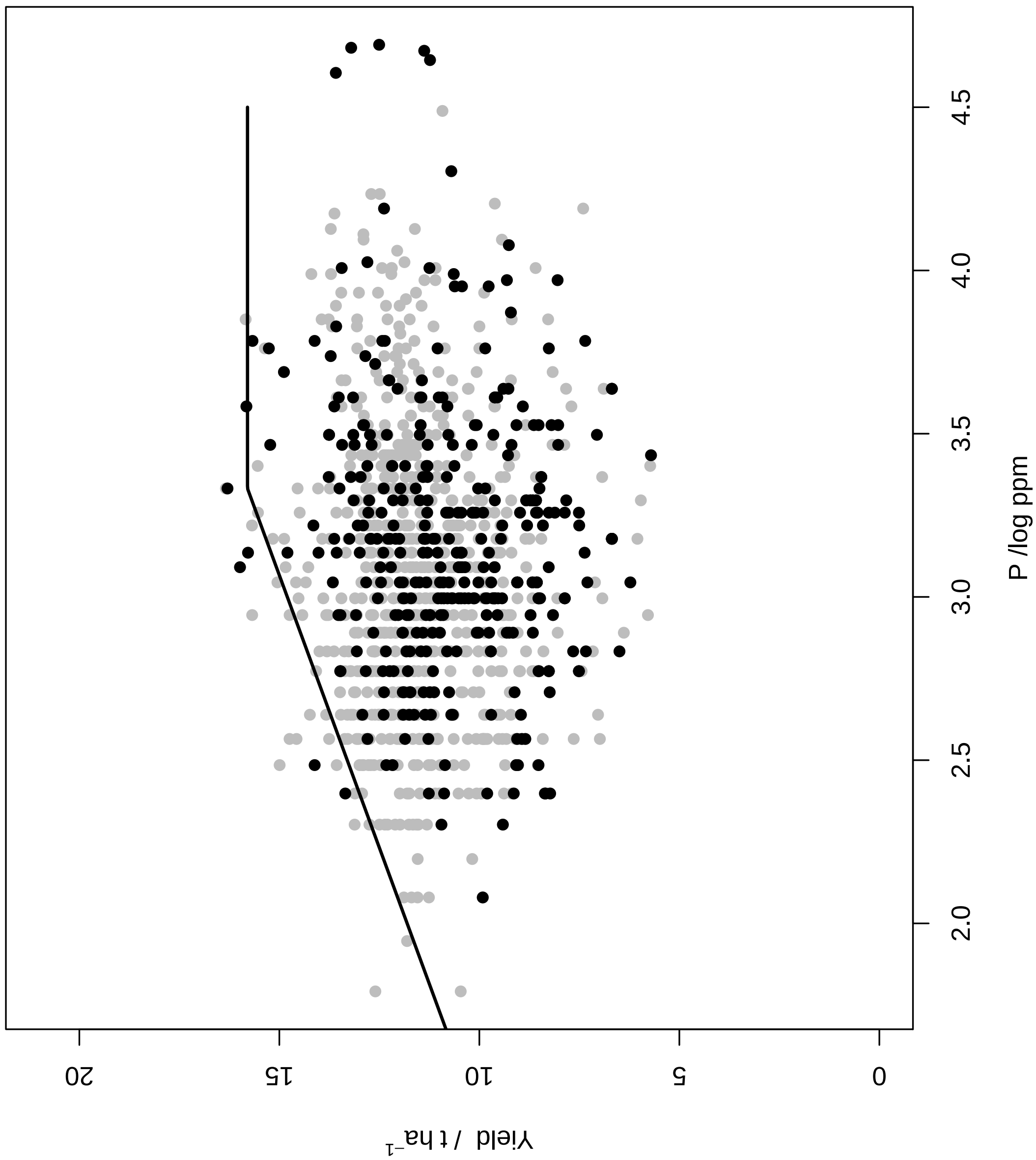

Supplementary Figure 15: Yield data (all wheat) from 2015 and soil P concentration and fitted boundary model for shallow soils (< 30 cm) with soils of pH≤ 8 shown in black and soils of pH> 8 in grey.
